# Brain topology underlying executive functions across the lifespan: focus on the default mode network

**DOI:** 10.3389/fpsyg.2024.1441584

**Published:** 2024-09-04

**Authors:** A. Menardi, M. Spoa, A. Vallesi

**Affiliations:** ^1^Department of Neuroscience, University of Padova, Padova, Italy; ^2^Padova Neuroscience Center, University of Padova, Padova, Italy; ^3^Department of General Psychology, University of Padova, Padova, Italy

**Keywords:** executing functions, resting state, default mode network, frontoparietal network, lifespan

## Abstract

**Introduction:**

While traditional neuroimaging approaches to the study of executive functions (EFs) have typically employed task-evoked paradigms, resting state studies are gaining popularity as a tool for investigating inter-individual variability in the functional connectome and its relationship to cognitive performance outside of the scanner.

**Method:**

Using resting state functional magnetic resonance imaging data from the Human Connectome Project Lifespan database, the present study capitalized on graph theory to chart cross-sectional variations in the intrinsic functional organization of the frontoparietal (FPN) and the default mode (DMN) networks in 500 healthy individuals (from 10 to 100 years of age), to investigate the neural underpinnings of EFs across the lifespan.

**Results:**

Topological properties of both the FPN and DMN were associated with EF performance but not with a control task of picture naming, providing specificity in support for a tight link between neuro-functional and cognitive-behavioral efficiency within the EF domain. The topological organization of the DMN, however, appeared more sensitive to age-related changes relative to that of the FPN.

**Discussion:**

The DMN matures earlier in life than the FPN and it ıs more susceptible to neurodegenerative changes. Because its activity is stronger in conditions of resting state, the DMN might be easier to measure in noncompliant populations and in those at the extremes of the life-span curve, namely very young or elder participants. Here, we argue that the study of its functional architecture in relation to higher order cognition across the lifespan might, thus, be of greater interest compared with what has been traditionally thought.

## 1 Introduction

Executive functions (EFs) are a family of higher order cognitive processes that facilitate goal-oriented thought and action (Anderson, 2001; Banich, 2009; Duncan and Owen, 2000; Norman and Shallice, 1986). They enable the coordination of behaviors that are considered characteristic of, although they are not unique to, humans (Goldberg, 2001), such as those linked to intentionality, inhibitory control and complex decision-making (Panikratova et al., 2020). While they have been traditionally localized to brain areas that are relatively immature until early adulthood (Menon and D’Esposito, 2021), core executive capacities have been elicited in children as young as 5 years (Anderson et al., 2002; Diamond, 2020; Espy, 2004). Given the rapid maturation of these higher order abilities in early life (Blair, 2016), their importance for optimal academic performance (Blair and Razza, 2007; Clark et al., 2010; Fuhs et al., 2014; Pascual et al., 2019) and behavioral control development (Schoemaker et al., 2013), as well as their role in predicting cognitive impairment in older age (Corbo and Casagrande, 2022), an understanding of the mechanisms underpinning executive integrity across the lifespan is at the forefront of neuroscientific research. Longitudinal neuroimaging studies are methodologically challenging (Skup, 2010), however, and findings from cross-sectional lesion-based studies have been largely inconsistent (Panikratova et al., 2020). Functional magnetic resonance imaging (fMRI) has, thus, gained popularity in identifying functional connectivity patterns in pediatric and elderly populations, particularly given its ease in signal acquisition and requirement of minimal effort (Smitha et al., 2017).

From a neuro-anatomical point of view, EFs appear supported by a distributed network of interconnected brain areas that include the dorsolateral prefrontal cortices (dlPFC), anterior cingulate cortices (ACC), posterior parietal cortices (PPC), supramarginal gyri (SMG), and inferior temporal gyri (ITG), collectively referred to as the frontoparietal network (FPN) (Marek and Dosenbach, 2018; Spreng et al., 2010). Joint activation between these regions has been regularly elicited in task-driven fMRI studies that have placed demands on selective attention (Chelazzi et al., 2011; Squire et al., 2013; Tang et al., 2021; Yantis, 2008) and, similarly, cognitive inflexibility during tasks has been associated with abnormal FPN connectivity (Liu et al., 2023). Importantly, the functional architecture of the FPN at rest has been associated with a general capacity to engage in goal-directed behavior even outside of the MRI environment (Reineberg et al., 2015). On the other hand, because of observed decreases in activity of the Default Mode Network (DMN) during overt attention-demanding tasks (Raichle, 2015), this network has traditionally been considered a hindrance for higher-order cognition (Spreng, 2012). Comprised of the ventromedial prefrontal cortices (vmPFC), the dorsomedial prefrontal cortices (dmPFC), posterior cingulate cortices (PCC), precuneus, superior temporal gyri (STG) and a portion of the lateral parietal cortex comprising the angular gyri (Raichle, 2015; Spreng et al., 2010), the DMN has been conceptualized as a network whose activity reflects stimulus-independent “baseline” conditions: those of daydreaming, mind wandering or emotional processing (Menon, 2023; Raichle, 2015; Spreng, 2012). Its activity, then, is considered negatively correlated—or “anticorrelated” (Fox et al., 2005)—with that of task-positive networks (e.g., the FPN), in which activity typically increases with increases in externally-driven cognitive demands. As such, the degree of suppression of the DMN during task-evoked activity has been reported as a trademark of healthy functioning, interpreted as a diminished risk of intrusion of irrelevant activity (e.g., mind wandering) that is traditionally supported by the DMN (for a review see Anticevic et al., 2012).

More recently, however, regions within the DMN alone have also been associated with different cognitive processes. The PCC has been causally implicated in episodic memory encoding (Natu et al., 2019), with abnormal activity in the region observed in mild cognitive impairment (Vanneste et al., 2021) and indicative of possible progression to Alzheimer’s disease (Lee et al., 2020), where it may even have a critical role in EF (Fu et al., 2023; Leech et al., 2011). The precuneus, on the other hand, has been found to have a causal role in the retrieval of episodic memories (Hebscher et al., 2020). Moreover, both the development of social cognition early in infancy (Grossmann, 2013), as well decision making processes (Csifcsák et al., 2021), appear at least partially regulated by the medial PFC, while the STG has been observed to play a role in multisensory integration, particularly of auditory and visual stimuli (Reale et al., 2007).

Interestingly, because of its distributed location in the cortex, the DMN is heavily connected to several sensory and association areas and may, thus, have a particularly important role as a control point for information processing, allowing otherwise segregated brain systems to be functionally connected (Buckner et al., 2009). A challenge for higher order cognition lies indeed in the integration of information. In line with this idea, a disproportionately drastic impact has been reported in cognitive functioning following damage to DMN hubs (Buckner et al., 2009). It might be reasonable, then, to reconsider the network architecture of the DMN itself in relation to higher order cognitive processes. For instance, several regions within the DMN (PCC, angular gyri, temporal lobes) have been reported to increase their connectivity with task-positive regions as a function of increased cognitive demands (task difficulty) (Cocchi et al., 2013; Hearne et al., 2015), challenging the view of the DMN and FPN as competing and functionally segregated networks (Dixon et al., 2018; Hasson et al., 2015; Kam et al., 2019; Katsumi et al., 2023; Raut et al., 2020; Zhang et al., 2019). Indeed, although both networks represent distinguishable entities with specific functions, their interplay is essential in high order cognitive functions (Cocchi et al., 2013; Menon and D’Esposito, 2021). As such, across-network interactions between the DMN and the FPN have been shown to be significant predictors of interindividual differences in executive functions (Hearne et al., 2015, 2016; Shi et al., 2018), with their inter-network connectivity progressively increasing during childhood (Chen et al., 2023) and decreasing with older age (Koshino et al., 2023).

Neuroimaging studies have traditionally investigated the association between brain and behavior through task-evoked fMRI paradigms. The use of tasks inside the scanner is challenging, however, as it suffers from inherent issues such as an increased risk of motion inside the scanner, as well as a dependence on a relatively high level of compliance from the participants to task demands (Fox and Greicius, 2010; Harms et al., 2018; Uddin et al., 2010). The latter aspect poses a particular challenge for studies across the lifespan, where part of the sample falls into the extremes of the aging curve; that is, younger children and older adults. Additional factors in favor of resting state relative to task-fMRI include: (i) a higher signal-to-noise ratio, given that over 80% of the signal in task-fMRI is discarded as noise, including spontaneous fluctuations that are correlated with specific resting state networks; (ii) a lower number of trials, since task-fMRI requires a high number of trials and extensive acquisition in order to derive reliable activation maps that can be difficult to achieve in noncompliant populations (e.g., very young or old participants); (iii) an opportunity to study multiple cortical regions at once, while task-fMRI requires dedicated protocols for each specific function or brain region (Fox and Greicius, 2010). Finally, the metabolism increase associated with a task is usually very small (<5%) compared to the high energy consumption associated with resting state activity (20% of the overall body metabolism), which leads to study task-related changes in brain activity between groups that often account for less than 1% of the signal (Fox and Greicius, 2010). Demonstrating that functional connectivity patterns at rest are equally associated with behavior outside of the scanner would, thus, prove beneficial in overcoming these issues. Because spontaneous fluctuations in the brain are known to self-organize in regions of activity—i.e., resting state networks (Biswal et al., 1997)—that mirror the activation patterns that are evoked by cognition (Cole et al., 2016), interindividual differences in intrinsic functional connectivity might be useful in predicting differences in brain activity that are task-evoked (Cole et al., 2016). This has recently been achieved by several studies in young healthy populations, proving that higher order cognitive functioning measured outside the scanner can be successfully predicted by regional connectivity that expands well beyond the FPN and involves a multitude of regions belonging to the DMN and attentional networks too (Hearne et al., 2016; Menardi et al., 2022; Reineberg et al., 2015). However, it is difficult to generalize those findings to younger and older age groups, given the diverse rate at which specific EF skills develop in the first years of life (McKenna et al., 2017), as well as the fact that EF are among the first cognitive functions to decline in the elderly population and are often accompanied by compensatory activity in the brain (Reuter-Lorenz et al., 2021). Lifespan studies are, hence, needed in trying to bridge this gap and to help determine the neural bases of EF as a function of age.

Paralleling changes in higher cognitive abilities, functional connectivity both within—and between—the FPN and DMN is subject to complex transformations across the lifespan (Edde et al., 2021). In line with the emergence of self-awareness in infants, the DMN undergoes significant and sustained development to achieve a well-distributed and adult-like network architecture within the first year of life (Gao et al., 2015). More specifically, an initial increase in within-network connectivity between the bilateral posterior regions of the DMN (i.e., medial temporal lobe and PCC) is followed by an increase in within-network connectivity between remote regions along the rostro-caudal axis (i.e., medial PFC and lateral temporal cortex). The FPN, by contrast, is characterized by a more progressive development, being one of the last functional networks to emerge (Chen et al., 2023; Edde et al., 2021; Kupis et al., 2021) and still relatively immature until well into adolescence (e.g., Liu et al., 2016). A reduction in within-network connectivity in both the FPN and DMN is observed to begin in middle age (Varangis et al., 2019), and becomes even more pronounced in older age (Farras-Permanyer et al., 2019). While this suggests an overall progressive age-related shift towards a more segregated functional architecture, topological changes in healthy aging have also been characterised by a more complicated pattern of both increases and decreases in connectivity between different networks (Zonneveld et al., 2019). Mixed results have, however, been reported in the literature on the association between connectivity and cognition (for a review, see Liem et al., 2021).

The present study aimed to investigate how changes in EF capacities across the lifespan might be related to brain organization as derived from resting-state data. Specifically, it was of interest to determine whether normal age-related changes in selective attention and cognitive flexibility—two core EF capacities—could be reliably understood in terms of functional topological alterations within the DMN and FPN across the lifespan. Building on prior literature, we tested (i) if resting state network topology might be associated with higher order cognitive abilities, (ii) how this relationship might be modulated by age, and (iii) whether a resting state network traditionally linked to baseline activity, the DMN, could show a higher association with EF compared to the traditional task-evoked activity network, the FPN. To address these aims, graph theory metrics representative of segregation and integration mechanisms in the brain were employed as main effects in the models, first simply to estimate the association between topological changes and age, and then to investigate how the topological properties of each network might be associated with outside-scanner EF performance as a function of age.

To the best of our knowledge, this is the first study to directly investigate the functional architecture of the DMN itself in relation to EFs across the lifespan.

## 2 Methods

### 2.1 Participants

Five hundred neurologically healthy (*n*_females_ = 267) participants were retrieved from the Human Connectome Project (HCP) Lifespan database,^1^ a rich and multi-model available set of consistently acquired neuroimaging and cognitive data, aimed at defining normative developmental and age-related changes in the brain (Harms et al., 2018). Participants were selected to maintain a balance between females and males from each age category, from 10 to 100 years of age (see Table 1).

**Table 1:** Summarizes the demographic characteristics of our sample.

Age (range)	Sex (n)	Race (%)	Ethnicity (%)	Handedness* mean (SD)
10–100 years	F (*n* = 267)M (*n* = 233)	Asian = 1.8%Black or African American = 9.8%Hawaiian or Pacific Islander = 10.2%More than one race = 5.8%Unknown or unreported = 2.4%White = 70%	Hispanic or Latino = 11.4%Not Hispanic or Latino = 88.2%Unknown = 0.4%	68.17(±46.9)

*Handedness was assessed using the 11-item Edinburgh Handedness questionnaire (Oldfield, 1971).

### 2.2 Cognitive measures

As part of the HCP fMRI protocol, participants were administered a broad neuropsychological test battery covering sensory, motor, emotion, memory, language and EF skills, with age-adjusted versions allowing for comparison across the lifespan. For the purpose of this study, however, only the Dimensional Change Card Sorting (DCCS) and the Flanker task were used as representative of EF functions, given that they were the only two EF tasks administered to all age cohorts. Scores obtained on a picture vocabulary (PVT) task were also used as a control to test the specificity of our findings. Further details on the administration and scoring of the tasks are openly available on the Human Connectome Project (HCP) Lifespan webpage (see footnote 1).

### 2.3 Neuroimaging measures

#### 2.3.1 fMRI data acquisition

Given the challenges pertinent to imaging developmental and aging populations, including an increased propensity for head movement (Geerligs et al., 2017; Satterthwaite et al., 2012) and a lower tolerance for long scan sessions (e.g., as a result of boredom in the younger participants or muscular distress in the older participants), a key decision was to limit the collection of data to an overall scanner acquisition time of 45 min per subject (Harms et al., 2018). This is slightly shorter relative to the traditional HCP protocols of 1 h per participant, but was considered an appropriate acquisition time given the age range of the sample. T1-weighted anatomical data [repetition time (TR) = 2,500 ms, time interval (TI) = 1,000 ms, matrix size = 320 × 300 × 208 mm, voxel size = 1 × 1 × 1 mm] and functional resting state data [acquisition parameters: number of volumes = 488, TR = 800 ms, echo time (TE) = 37 ms, voxel size = 2 × 2 × 2 mm, flip angle (FA) = 52 deg.] were collected according to the HCP Lifespan acquisition protocols (Harms et al., 2018). The data provided had already undergone HCP minimal preprocessing steps (Glasser et al., 2013), including correction for spatial distortion, motion, bias field, and surface registration. Temporal artifacts were cleaned from the data using independent component analysis and a machine learning classifier (FIX) (Glasser et al., 2016). Only spatial smoothing using a Gaussian kernel of FWHM 6.0 mm was further applied. A quality check (QC) on the extent of head motion inside the scanner was performed by looking at the individual average framewise displacement across all scans (range: 0.61 mm—0.02 mm; mean = 0.2 mm, std. = 0.09 mm), proving good quality of the data. Indeed, the average absolute value remained below half a voxel width, which is generically considered a threshold to determine the quality of the data with respect to head motion (Power et al., 2012). However, we observed a weak positive relationship between age and head motion (*r* = 0.19, *p* < 0.0001). Additional QC controls are applied as part of the HCP Lifespan processing pipeline, both in real-time during data acquisition, as well as by means of post-acquisition manual and automated QC. An extensive list of all QC procedures is available in the published work by Marcus et al. (2013). In summary, structural scans are manually inspected by an expert rater and any anomaly further evaluated by experienced neuroradiologists (Elam et al., 2021). If brain abnormalities are present which could affect brain connectivity estimates, the participant’s data (including his/her behavioral data) are not released (Elam et al., 2021). Further QC on the structural data is performed following Freesurfer surface reconstruction to readily evaluate grey and white matter segmentations (Elam et al., 2021). As for the functional data, another set of QC pipelines is used to determine signal-to-noise ratios and flag motion outliers (Elam et al., 2021). Individuals’ data with excessive head motion are also not made available in the release (Elam et al., 2021). This might come with a caveat, in the sense that all individuals who completed the protocol and provided high quality data, as made available by from the HCP, might not be representative of their age category, but rather represent a “super-normal” sample (e.g., for older participants) (Bookheimer et al., 2019).

Finally, individual brains were parcelled into 200 regions of interest (ROIs) according to the Schaefer et al. (2018), and functional connectivity matrices were computed from the individual time series by correlating the BOLD signal of every pair of ROIs. Individual connectivity matrices were then further transformed into Fisher’s z-scores to ensure normality, and to ease the comparison and the interpretability of connectivity estimates (Yu et al., 2018). Our analyses were also run using the 400 regions parcellation scheme of the Schaefer et al. (2018), available in the Supplementary material.

### 2.4 Graph theory

In order to define changes in communication efficiency between components of the FPN (consisting of 30 nodes) and the DMN (consisting of 46 nodes) across the lifespan, and to understand how these might be associated with cognitive performance, the present study employed graph theory principles (Bullmore and Sporns, 2009). First, a false discovery rate (FDR) correction (*𝛼*= 0.05) was applied to all connectivity matrices to reduce the risk of false positives while ensuring that sufficient interindividual variability was kept (Bassett et al., 2006; Drakesmith et al., 2015), allowing us to retain the 80% of the edges on average. While traditional approaches to thresholding have typically employed arbitrary cut-offs as a means of retaining the strongest 10–40% of edges (Bullmore and Bassett, 2011), such methods are highly conservative and can bias the topological properties of a network (Drakesmith et al., 2015). Indeed, high thresholding approaches tend to result in relatively disconnected networks that are not representative of a true connectome (Drakesmith et al., 2015).

A more permissive single statistical threshold application by means of FDR or Bonferroni correction to the single correlations in the connectivity matrices can reduce the risk of both Type-I and Type-II error rates, while retaining interindividual variability (Cao et al., 2014). Such an approach appears particularly relevant in the context of developmental research where, despite an apparent connectome macrostructure by the age of 2 years, rearrangements at a more micro level (e.g., edge weights) continue to occur throughout the lifespan (Collin and Van Den Heuvel, 2013). It was therefore considered appropriate to threshold the matrices in a manner that would preserve as much of this latter source of variability as possible in the present study. Adjacency matrices were derived for each individual, in which the a_ij_ elements were equal to the Fisher-z transformed value of the correlation between nodes i and j, or zero otherwise (i.e., in the absence of a connection).

Graph theory measures were extracted via the Brain Connectivity Toolbox^2^ function running in MATLAB 2023a (The Mathworks, Inc., Natick, MA, United States). Measures of integration (i.e., Characteristic Path Length, Global Efficiency), segregation (i.e., Clustering Coefficient and Modularity), and their balance within the system (Small Worldness) were extracted separately for both the FPN and the DMN to allow a network-level analysis, and were defined as follows:

Integration measures:

i) Characteristic Path Length (CP): the average distance between a node and all the other nodes of the system;
ii) Global Efficiency (GE): the inverse of the average shortest path, that is, the average of the efficiency over all pairs of nodes.

Segregation measures:

iii) Clustering Coefficient (CC): the fraction of nodes being neighbors with the surrounding nodes, forming triangular triplets;
iv) Modularity (MOD): the extent to which a network can be divided into distinct modules based on greater within-module, rather than between-modules, edges.

And their balance:

v) Small-Worldness (SW): the property of a system to have a concomitant high clustering coefficient and a low path length.

Because the FPN and the DMN can be divided into anatomically distinct components, nodes were defined within both the FPN and the DMN to better estimate their contributions to cognitive changes across the lifespan. In particular, parcellations were based on the Schaefer et al. (2018), such that the FPN was subdivided into 30 nodes and the DMN into 46 nodes. Separate topological measures were extracted for the individual regions, and were defined as follows:

i) Characteristic Path Length (CP): the average distance of a given region to all the other nodes of the network;
ii) Clustering Coefficient (CC): the tendency of a given region to show a greater distribution of edges towards neighboring nodes, forming triangular triplets;
iii) Betweenness Centrality (BC): the fraction of all shortest paths that pass through a given region;
iv) Nodal Degree (ND): the number of connections of a given region.
v) Participation Coefficient (PC): a measure of diversity of intermodular connections of the individual nodes.

For an in-depth explanation of graph theory measures, see Rubinov and Sporns (2010).

### 2.5 Data analysis plan

Data were analyzed using MATLAB 2023a (The Mathworks, Inc., Natick, MA, United States). To assess topological changes at the network level as a function of age, linear correlation analyses were performed between the aforementioned graph theory measures and age, separately for the FPN and the DMN. To minimize the risk of Type-I error due to multiple comparisons, the results of all models were corrected using False Discovery Rate (FDR), with statistical significance considered against *𝛼* = 0.05.

Then, multiple linear regression models were employed to determine if brain topology at the network level, as indexed by graph theory measures, was associated with EF abilities, operationalized by performance on the Flanker and the DCCS tasks. Performance on these tasks was analyzed separately as they reflect different components within the EF framework: selective attention/inhibition and cognitive flexibility, respectively. Of particular interest were the possible interactions between age and the functional properties of each network in regard to higher order cognitive performance and, thus, age was included as an interaction term in each of the models. Moreover, because developmental trajectories for executive abilities have been found to differ slightly as a function of sex (De Luca et al., 2003), sex was controlled for. Given the exploratory nature of the study, we did not want to impose any *a priori* assumption on the influence of age on the model and so both linear and quadratic effects were tested. A Likelihood Ratio Test was then used to determine the model with the better fit. To minimize the risk of Type-I error, only models surviving FDR correction (𝛼 = 0.05) were considered. Furthermore, all analyses were controlled for potential head motion effects, measured as the individual mean of the framewise displacement across all scans.

The same multiple regression models were employed at the node level of each network, with the node-specific graph theory measures evaluated as factors in the analyses. The variance inflation factor (VIF) was computed to ensure that only factors with no risk of multi-collinearity were inserted in the models, resulting in the choice of CP, MOD and SW as factors for the analyses conducted at the network level and CP, CC and BC as factors for the analyses conducted at the node level. Outliers were removed based on the models’ residuals, as determined by a cut-off of ±3 scaled absolute deviations from the median.

At last, we estimated the extent to which each node of the DMN and of the FPN preferentially connects to nodes of the same community (DMN-DMN, FPN-FPN) or across communities (DMN ↔ FPN) by evaluating their measures of PC. We then tested the association between the average value of PC for each network (DMN and FPN, respectively) and EF measures in both linear and quadratic fashion.

## 3 Results

To get a general idea about the nature of topological change at the network level in association with age, linear correlations were run between graph theory measures and age for both the FPN and the DMN. Multiple regression analyses were then used to evaluate the strength of the association between topological properties and EF in consideration of age; first at a network level, to get a global picture of network characteristics associated with cognitive function and, subsequently, at a node level, to evaluate how EF development and decline over the lifespan might be a product of the age-affected topographical properties of specific anatomical components within each of the networks. For each model, we performed an analysis of the standard residuals and observed an average of 6 outliers, which were then removed. We also checked for the strength of the association between EF and age in their linear (Flanker; *r* = 0.17, *p* = 0.0002, R^2^ = 0.03; DCCS: *r* = 0.23, *p* < 0.0001, R^2^ = 0.054) and quadratic (Flanker: *F*_(2,475)_ = 7.27, *p* < 0.0001, R^2^ = 0.03); (DCCS: F_(2,475)_ = 14.8, *p* < 0.0001, R^2^ = 0.058) relationships, observing weak positive associations.

Finally, control analyses were run on a network whose connectivity was not expected to correlate with EF abilities, i.e., the sensorimotor network (see Supplementary materials).

### 3.1 FPN

#### 3.1.1 Network-level analyses

**Figure 1:**
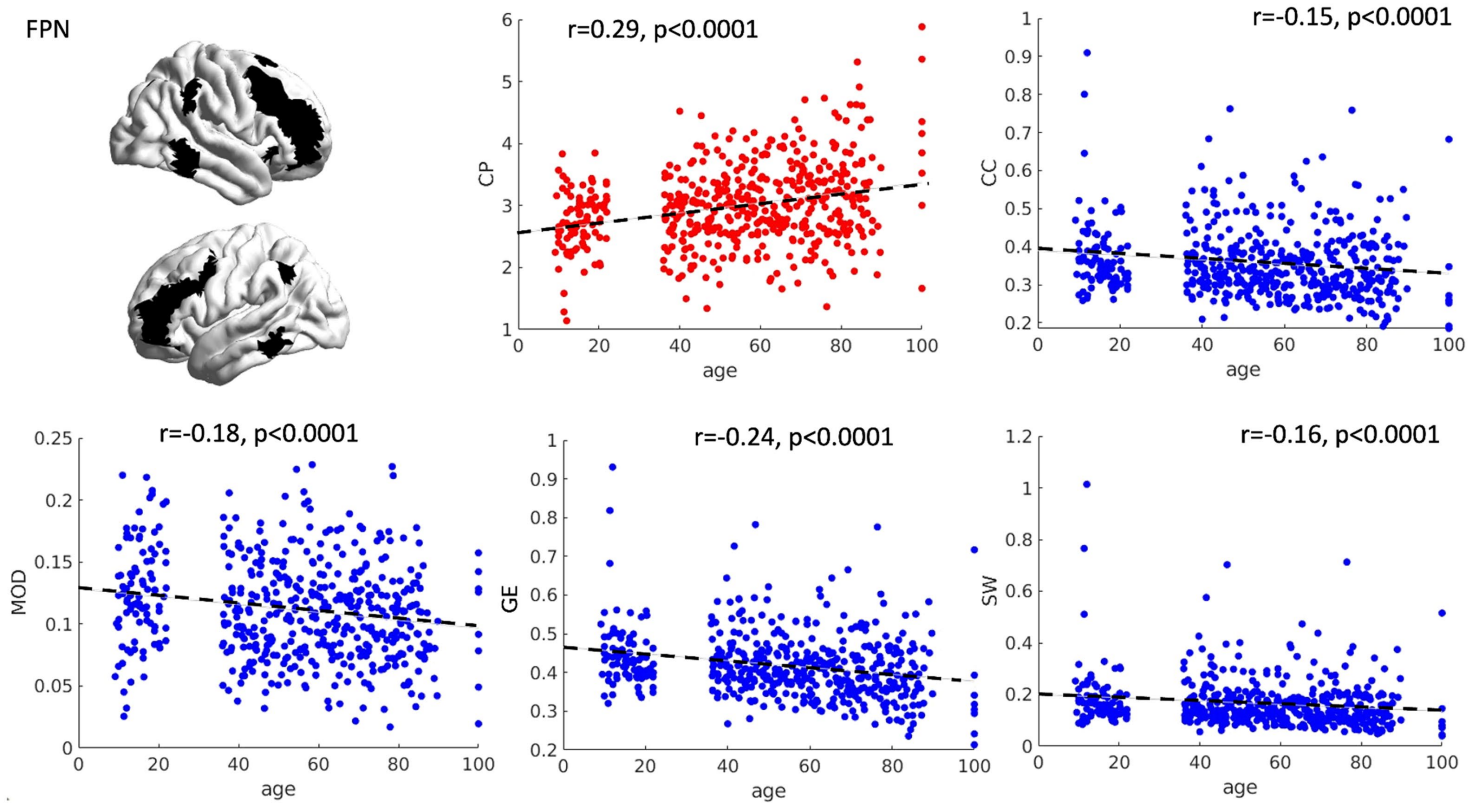
Scatterplots of network topology with age. The relationships between age and the topology of the FPN is presented as scatterplots. As a function of age, the FPN shows a progressive loss of connection strength (higher characteristic path length—CP), as well as reduced segregation and specialization of the networks (lower clustering coefficient and modularity). Furthermore, a general reduction in network efficiency was observed (lower global efficiency and small worldness). CP = characteristic path length, CC = clustering coefficient, MOD = modularity, GE = global efficiency, SW = small worldness.

The relationship between age and graph theory measures was investigated by means of correlation analyses, revealing a significant positive association between age and CP (*r* = 0.29, *p* < 0.0001) and a significant negative relationship between age and CC (*r* = −0.15, *p* < 0.0001), GE (*r* = −0.24, *p* < 0.0001), MOD (*r* = −0.18, *p* < 0.0001) and SW (*r* = −0.16, *p* < 0.0001) (see Figure 1), suggesting an increased distance and loss of network specialization and efficiency as a function of age. Notably, we also tried to fit the relationship between age and graph theory measures in a quadratic fashion. However, almost identical fits and R^2^ values were obtained, suggesting that the relationship between our variables is already captured at the linear level.

The overall multiple regression models, which considered age, gender, head motion and FPN topology, as well as their interaction, in association with Flanker and DCCS performance emerged as significant (Flanker: R^2^ = 0.08; *F*_(12, 459)_ = 3.3, *p* = 0.0001); DCCS: (R^2^ = 0.08, *F*_(12, 464)_ = 3.61, *p* < 0.0001). For both models, age emerged as significantly associated with cognitive performance (Flanker: *β* = 2.78, *p* < 0.0001, DCCS: *β* = 5.32, *p* < 0.0001), suggesting that variability in the performance scores can be related to differences in age rather than topological properties of the FPN. The individual amount of motion inside the scanner was also observed to be negatively correlated with performance on the Flanker task (*β* = −16.6, *p* = 0.012), suggesting that the individuals with less head movement were also the ones who had higher performance scores as assessed outside the scanner. To make sure that our results were not biased by potential movement confounds, we checked for the interaction between our graph theory measures and framewise displacement, which were not significant.

The model testing the association between FPN topology and performance on the PVT, our control task, was not significant (R^2^ = 0.02, *F*_(12,461)_ = 0.95, *p* = 0.493).

In addition, we tested if a simpler measure of within-network connectivity strength could be significantly associated with EF performance across the lifespan in either a linear or quadratic fashion. We observed no significant correlation for neither the Flanker (linear: *r* = 0.03, *p* = 0.53; quadratic: F_(2,475)_ = 0.43, *p* = 0.64), nor the DCCS task (linear: *r* = −0.02, *p* = 0.59; quadratic: F_(2,475)_ = 0.14, *p* = 0.86).

#### 3.1.2 Node-level analyses

To analyze the differential relationship between FPN components, or nodes, and high order performance, the same analyses were conducted separately for each region. Overall, the topological properties of five FPN regions were significantly associated with selective attention (operationalized as performance on the Flanker task), and the topological properties of two FPN regions were significantly associated with cognitive flexibility (operationalized as performance on the DCCS). Importantly, significant interactions were observed between FPN topology and age, such that the relationship between the functional architecture of the FPN and EF performance varied depending on the age of the individual in either a linear or quadratic fashion.

**Figure 2:**
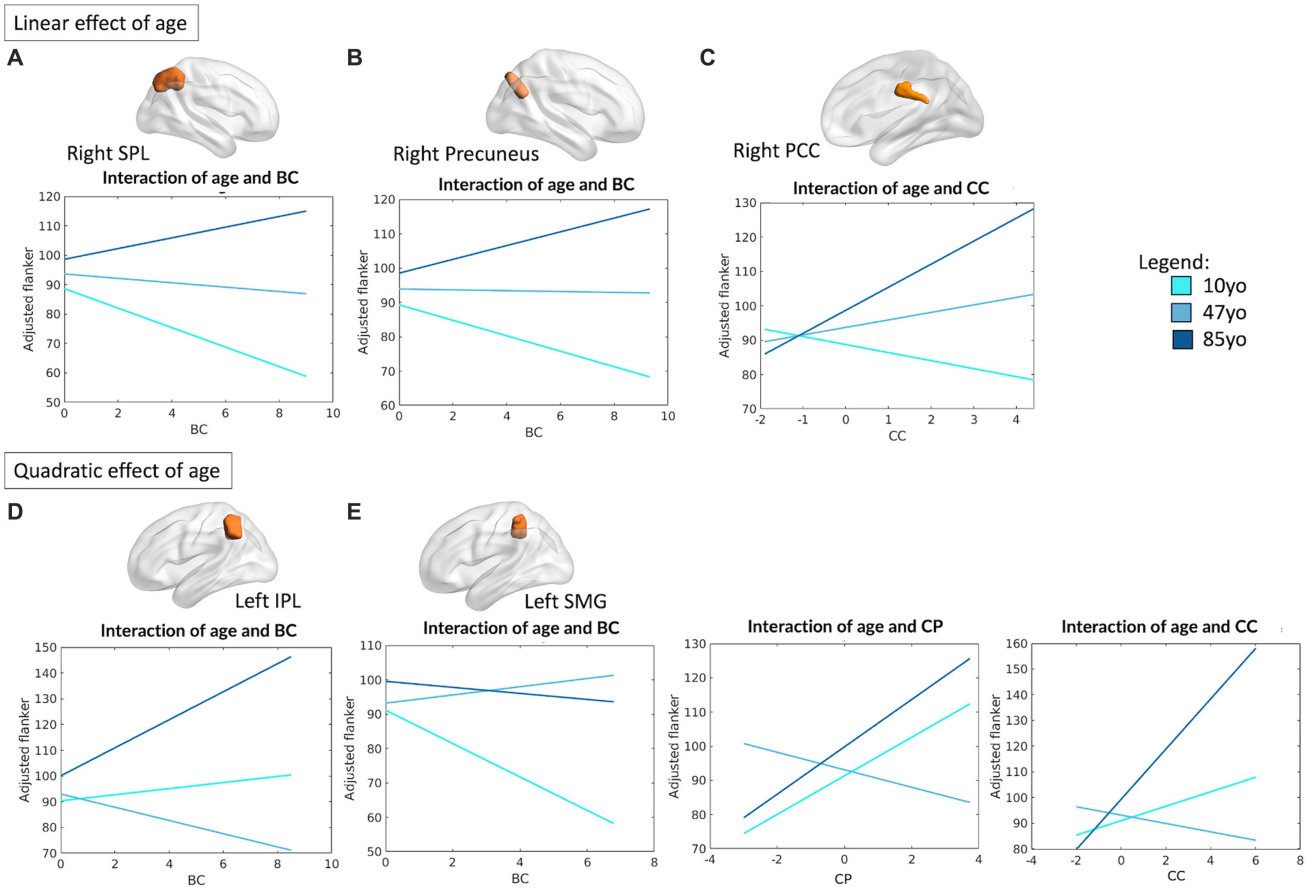
Linear and quadratic interactions between FPN topology and age in Flanker performance. Significant linear interactions between age and graph theory measures associated with Flanker performance are presented graphically for the right SPL **(A)**, right precuneus **(B)**, right PCC **(C)**. On the other hand, significant quadratic interactions between age and graph theory measures associated with Flanker performance were observed for the left IPL **(D)** and the left SMG **(E)**. Age is subdivided into three categories for interpretability, with the average age of each group shown: 10 yo = 10 years old; 48 yo = 48 years old; 85 yo = 85 years old. BC = betweenness centrality, CC = clustering coefficient, CP = characteristic path length, IPL = inferior parietal lobule, SMG = supramarginal gyrus, SPL = superior parietal lobule, PCC = posterior cingulate cortex.

Specifically, the right superior parietal lobule (SPL) (R^2^ = 0.07, *F*_(12,459)_ = 2.86, *p* = 0.0008) and right precuneus (R^2^ = 0.09, *F*_(12,459)_ = 3.74, *p* < 0.0001) were observed to be meaningfully associated with performance on the Flanker task, all showing significant linear interactions between their measure of BC and age (right SPL: *β* = 1.56, *p* = 0.043; right precuneus: *β* = 1.29, *p* = 0.023). For both regions, an increased centrality within the network was positively associated with performance in the older individuals, with an opposite relationship in the younger counterpart instead (see Figures 2A,B). The right PCC was also meaningfully associated with performance on the Flanker task (R^2^ = 0.09, *F*_(12,459)_ = 3.81, *p* < 0.0001), with a positive interaction between CC and age (*β* = 2.74, *p* = 0.032), suggesting that higher CC is associated with better performance in the middle and older age groups, with an opposite trend in the younger counterpart (see Figure 2C).

On the other hand, the topological properties of both the left inferior parietal lobule (IPL) (R^2^ = 0.09, *F*_(16,455)_ = 2.91, *p* = 0.0001) and left SMG (R^2^ = 0.10, *F*_(16,455)_ = 3.24, *p* < 0.0001) emerged as significantly associated with Flanker performance, with significant quadratic interactions between their measure of BC and age (left IPL: *β* = 2.17, *p* = 0.003; left SMG: *β* = −1.49, *p* = 0.028). For the left SMG, meaningful quadratic relationships were also observed between age and its measure of CP (*β* = 3.26, *p* = 0.017) and CC (*β* = 2.92, *p* = 0.022). Overall, this suggests the presence of a “U” shaped relationship between the measures of CP and CC of the left SMG and individual performance on the Flanker tasks, such that both younger children and older adults seem to benefit from increased local processing of the information, whereas the opposite is observed for middle-aged individuals. While the same “U” shaped relationship applies for the BC of left IPL—such that younger and older individuals benefit from a higher centrality of this region—an inverted “U” shape best depicts the relationship between BC and Flanker performance for the left SMG. In this case, a less central role of this region ensures better performance in younger and older individuals. A visual depiction of the aforementioned interactions is presented in Figures 2D,E.

Irrespective of age, significant associations between CC and performance on the Flanker task were observed for the left middle frontal gyrus (MFG) (R^2^ = 0.08, *F*_(12,459)_ = 3.46, *p* < 0.0001; *β* = 5.85, *p* = 0.047), the left DLPFC (R^2^ = 0.07, *F*_(12,459)_ = 3.14, *p* = 0.0002; *β* = 7.06, *p* = 0.032) and the right MFG (R^2^ = 0.08, *F*_(12,459)_ = 3.34, *p* = 0.0001; *β* = 7.24, *p* = 0.023). The CP of the right MFG was also observed to be positively associated with Flanker performance (*β* = 8.29, *p* = 0.025).

For all the nodes, motion emerged as a significant main effect in association with Flanker performance (left IPL: *β* = −17.4, *p* = 0.007; left SMG: *β* = −17.38, *p* = 0.006; left MFG: *β* = −18.09, *p* = 0.006; left DLFPC: *β* = −16.17, *p* = 0.012; right SPL: *β* = −16.52, *p* = 0.01; right MFG: *β* = −18.22, *p* = 0.004; right precuneus: *β* = −18.72, *p* = 0.004; right PCC: *β* = −21.19, *p* = 0.001).

**Figure 3:**
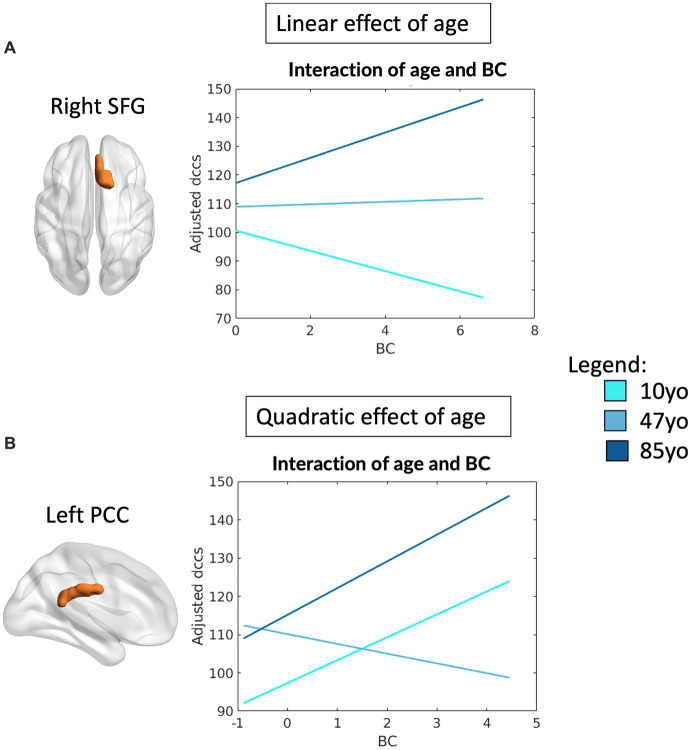
Linear and quadratic interactions between FPN topology and age in DCCS performance. Significant linear interactions between age and graph theory measures associated with DCCS task performance were observed for the right SFG **(A)**. On the other hand, the left PCC presented a quadratic effect of age on the relationship between topology and DCCS performance **(B)**. Age is subdivided into three categories for interpretability, with the average age of each group shown: 10 yo = 10 years old; 48 yo = 48 years old; 85 yo = 85 years old. DCCS = Dimensional Change Card Sorting task, SFG = superior frontal gyrus, PCC = posterior cingulate cortex, BC = betweenness centrality.

As for what concerns performance on the DCCS, the topological profile of two FPN regions showed significant interactions as a function of age: the right superior frontal gyrus (SFG) (R^2^ = 0.08, *F*_(12, 464)_ = 3.43, *p* < 0.0001) and left PCC (R^2^ = 0.09, *F*_(16, 460)_ = 3.04, *p* < 0.0001). In particular, significant interactions were observed between the BC of these regions and performance on the DCCS, which were observed to be linear for the right SFG (*β* = 2.4, *p* = 0.026) and quadratic for the left PCC (*β* = 3.33, *p* = 0.001). A significant main effect of head motion was observed for the left PCC (*β* = −18.12, *p* = 0.044). Figure 3 reports all significant interactions between topology and age associated with DCCS performance for both regions. Overall, these results highlight the importance of centrality of two main regions within the FPN, suggesting that increased centrality of the left PCC favors both elder and younger individuals, whereas increased centrality of the right SFG benefits elders but disfavors performance of the younger participants.

**Figure 4:**
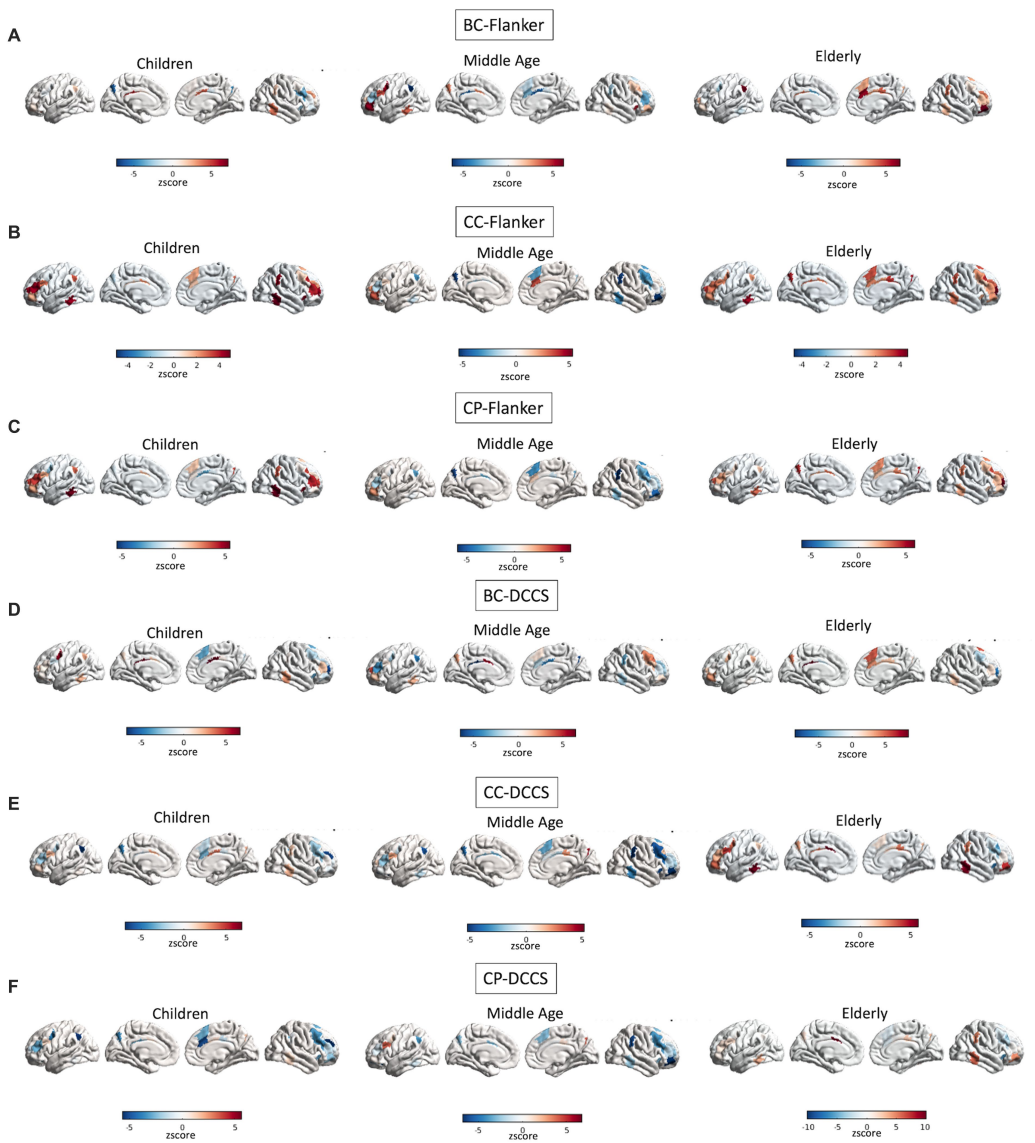
Interactions between age and topology associated with EF performance. Quadratic relationships are shown between topology of all regions of the FPN and performance at the Flanker **(A–C)** and DCCS **(D–F)** tasks. Colors reflect the slope of the interaction in z scores, such as that cooler colors reflect more negative relationships whereas warmer colors reflect more positive relationships.

A full depiction of the relationship between age and topology of all of the network’s regions associated with EF performance is shown in Figure 4.

### 3.2 DMN

#### 3.2.1 Network-level analyses

**Figure 5:**
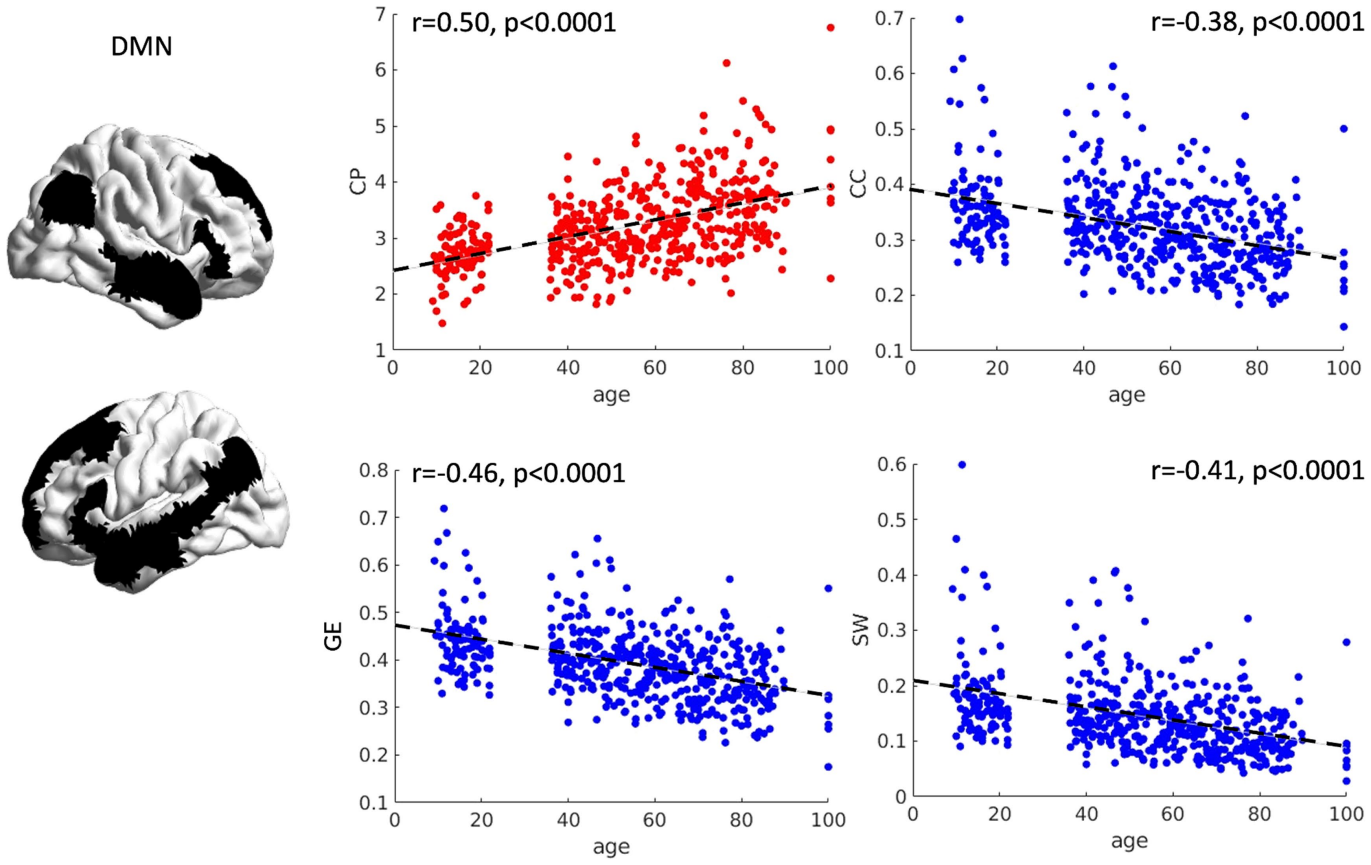
Scatterplots of network topology with age. The relationships between age and the topology of the DMN is presented as scatterplots. As a function of age, the DMN shows a progressive loss of connection strength (higher characteristic path length), as well as reduced segregation (lower clustering coefficient). Furthermore, a general reduction in network efficiency was observed (lower global efficiency and small worldness). CP = characteristic path length, CC = clustering coefficient, GE = global efficiency, SW = small worldness.

The relationship between age and graph theory measures was also investigated for the DMN by means of correlation analyses. Significant correlations were observed between CP (*r* = 0.50, *p* < 0.001), CC (*r* = −0.38, *p* < 0.001), GE (*r* = −0.46, *p* < 0.001) and SW (*r* = −0.41, *p* < 0.001) with age, suggesting a significant decrease in efficiency and loss of network specialization as a function of age (see Figure 5). Notably, we also tried to fit the relationship between age and graph theory measures in a quadratic fashion. However, almost identical fits and R^2^ values were obtained, suggesting that the relationship between our variables is already captured at the linear level. Because we observed higher correlation coefficients in the DMN compared with the FPN (see section 3.1.1.), we decided to compare them statistically using the package cocor (Diedenhofen and Musch, 2015). We observed that correlations between graph theory measures and age in the DMN were significantly higher than those observed for the FPN (CP: *z* = 7.01, *p* < 0.0001; CC: *z* = −7.18, *p* < 0.0001; GE: *z* = −7.43, *p* < 0.0001; MOD: *z* = 3.06, *p* = 0.002; SW: *z* = −7.95, *p* < 0.0001).

The overall multiple regression model, which considered age, gender, head motion and DMN topology, as well as their interaction, was significantly associated with both Flanker (R^2^ = 0.11, *F*_(12,459)_ = 4.89, *p* < 0.0001) and DCCS (R^2^ = 0.09, *F*_(12,464)_ = 4.12, *p* < 0.0001) performance. In particular, for both tasks, we observed a main effect of MOD (Flanker: *β* = −6.66, *p* = 0.0001; DCCS: *β* = −6.22, *p* = 0.012) and age (Flanker: *β* = 1.6, *p* = 0.042; DCCS: *β* = 3.77, *p* = 0.0007). Overall, this suggests that better performance on the Flanker and DCCS tasks is based on a reduced modularity within the DMN, possibly highlighting a benefit when the topology of the DMN shows less segregation and higher integration between its nodes. Notably, a main effect of motion was observed for the Flanker task (*β* = −15.9, *p* = 0.017), suggesting that individuals with higher head motion performed worse in the cognitive evaluation outside the scanner. No significant interaction between our graph theory measures and head motion was observed.

The model testing the association between DMN topology and performance on the PVT, our control task, emerged as not significant (R^2^ = 0.02, *F*_(12, 462)_ = 0.91, *p* = 0.532).

In addition, we tested if a simpler measure of within-network connectivity strength could be significantly associated with EF performance across the lifespan in either a linear or a quadratic fashion. We observed no significant correlation for the Flanker task (linear: *r* = −0.01, *p* = 0.78; quadratic: *F*_(2,475)_ = 0.25, *p* = 0.77). On the other hand, a significant linear negative association with the DCCS task was observed (*r* = −0.10, *p* = 0.02), but no quadratic association (F_(2,475)_ = 2.7, *p* = 0.07) (see Supplementary Figure S5).

#### 3.2.2 Node-level analyses

**Figure 6:**
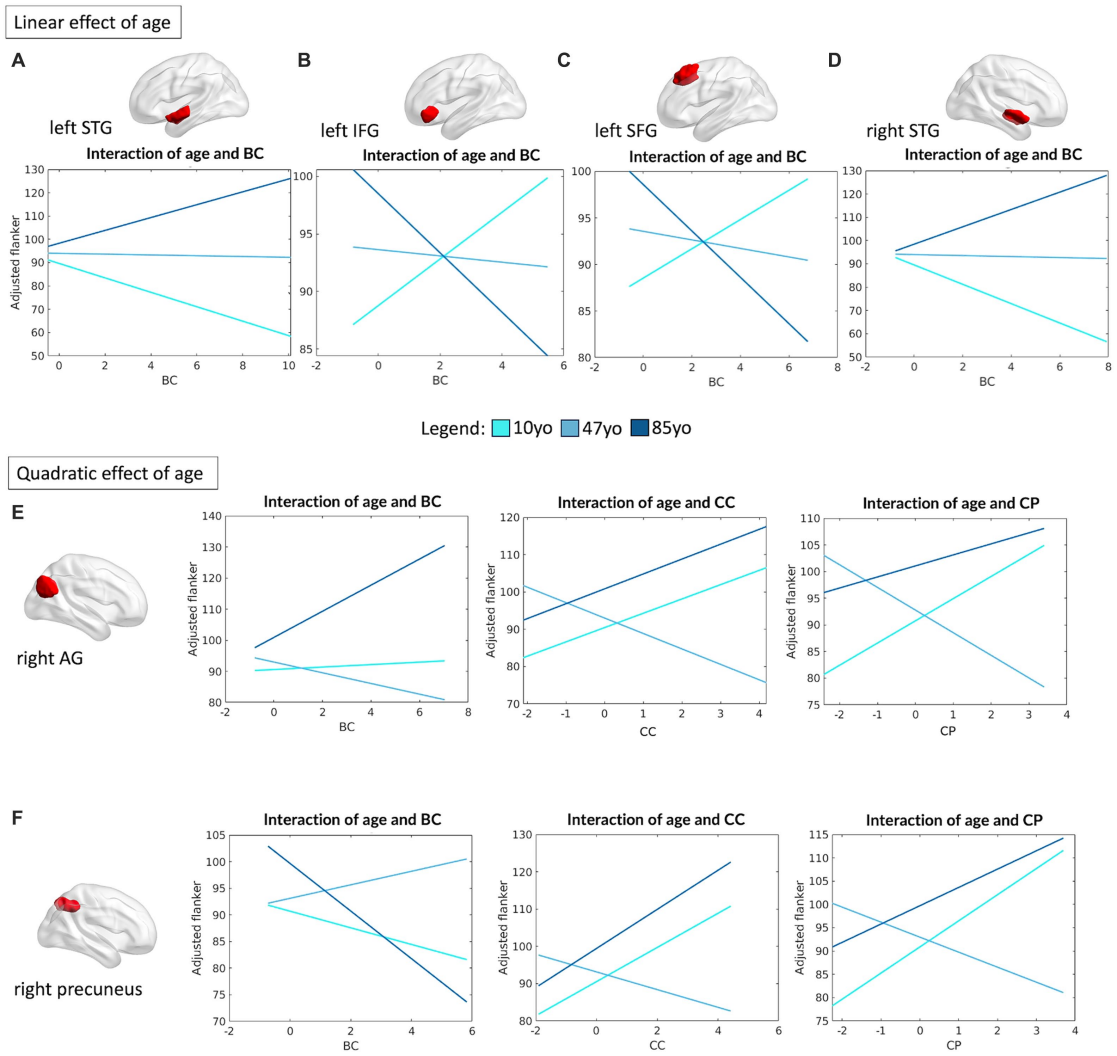
Linear and quadratic interactions between DMN topology and age in Flanker performance. Significant linear interactions between age and graph theory measures associated with Flanker performance are presented graphically for the left STG **(A)**, left IFG **(B)**, left SFG **(C)** and right STG **(D)**. On the other hand, significant quadratic interactions between age and graph theory measures associated with Flanker performance were observed for the right AG **(E)** and the right precuneus **(F)** for measures of BC, CC ad CP, respectively. Age is subdivided into three categories for interpretability, with the average age of each group shown: 10 yo = 10 years old; 48 yo = 48 years old; 85 yo = 85 years old. AG = angular gyrus, BC = betweenness centrality, CC = clustering coefficient, CP = characteristic path length, IFG = inferior frontal gyrus, SFG = superior frontal gyrus, STG = superior temporal gyrus.

To determine if specific regions within the DMN might drive the interaction between network topology and cognitive performance as a function of age, the same analyses were repeated at the single region level. In this regard, four regions of the DMN were found to be associated with individual selective attention abilities (operationalized by Flanker performance), with a meaningful linear interaction between age and their topological profile. Specifically, the left superior temporal gyrus (STG) (R^2^ = 0.07, *F*_(12,459)_ = 3.07, *p* = 0.0003), left IFG (R^2^ = 0.09, *F*_(12,459)_ = 3.89, *p* < 0.0001), left superior frontal gyrus (SFG) (R^2^ = 0.07, *F*_(12,460)_ = 3.19, *p* = 0.0002) and right STG (R^2^ = 0.09, *F*_(12,459)_ = 3.85, *p* < 0.0001) all showed a significant interaction between their degree of centrality in the network and Flanker performance (left STG: β = 1.78, *p* = 0.019; left IFG: *β* = −1.4, *p* = 0.011; left SFG: *β* = −1.23, *p* = 0.034; right STG: *β* = 2.39, *p* = 0.001). Interestingly, the left and right STG showed similar patterns, with a negative impact of their degree of centrality on Flanker performance in young children, but a favorable (positive) effect in the older adults. Conversely, a greater BC of the left IFG and left SFG appeared beneficial in younger children, but not in the older group (Figures 6A–D).

More complex interaction patterns were observed for the right angular gyrus (AG) (R^2^ = 0.09, *F*_(16,455)_ = 2.88, *p* = 0.0001), which showed meaningful quadratic interactions between its degree of BC (*β* = 1.48, *p* = 0.045), CC (*β* = 2.97, *p* = 0.017) and CP (*β* = 2.71, *p* = 0.045) and age in association with Flanker performance. The same relationships were observed for the right precuneus (R^2^ = 0.10, *F*_(16,455)_ = 3.13, *p* < 0.0001): BC (*β* = −1.58, *p* = 0.013), CC (*β* = 2.7, *p* = 0.041) and CP (*β* = 2.94, *p* = 0.045). For both regions, the degree of CP and CC were positively associated with performance at the Flanker task at the extremes of the aging curve (i.e., younger children and older adults), but negatively associated in middle aged individuals. On the other hand, the centrality of the right AG was observed to be strongly positively associated with attention performance in older adults, but with little or no relationship in the younger age groups. The centrality of the right precuneus was instead negatively associated with performance in the young and older groups, but positively in the middle-aged group (see Figures 6E,F).

All regions also showed a significant main effect of head motion (left STG: *β* = −17.22, *p* = 0.008; left IFG: *β* = −17.7, *p* = 0.006; left SFG: *β* = −17.55, *p* = 0.008; right AG: *β* = −15, *p* = 0.022; right STG: *β* = −18.6, *p* = 0.003; right precuneus: *β* = −16.6, *p* = 0.013).

**Figure 7:**
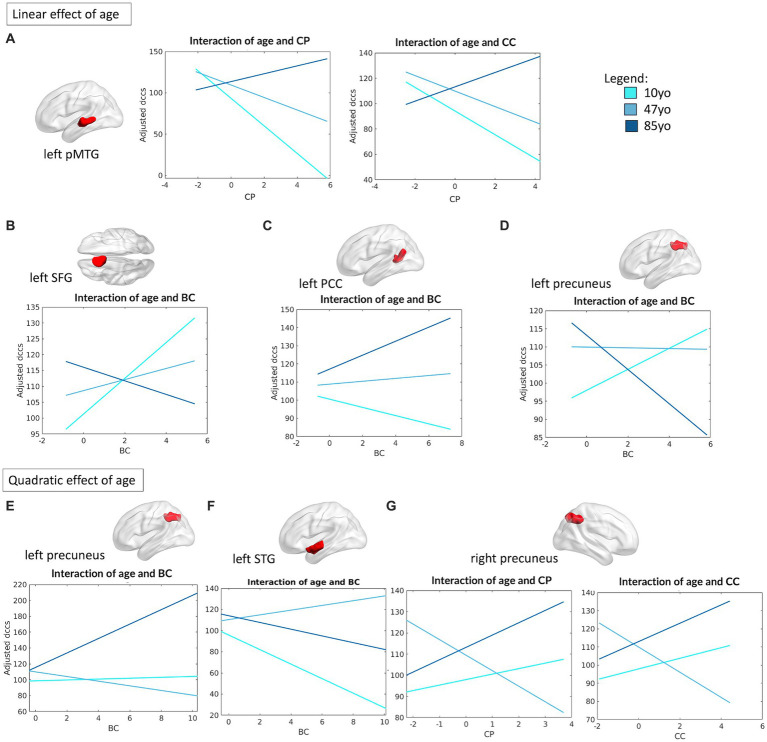
Linear and quadratic interactions between DMN topology and age in DCCS performance. Significant linear interactions between age and graph theory measures associated with DCCS performance are presented graphically for the left pMTG **(A)**, left SFG **(B)**, left PCC **(C)** and left precuneus **(D)**. On the other hand, significant quadratic interactions between age and graph theory measures associated with DCCS performance were observed for the left precuneus **(E)**, left STG **(F)** and the right precuneus **(G)**. Age is subdivided into three categories for interpretability, with the average age of each group shown: 10 yo = 10 years old; 48 yo = 48 years old; 85 yo = 85 years old. BC = betweenness centrality, CC = clustering coefficient, CP = characteristic path length, pMTG = posterior middle temporal gyrus, PCC = posterior cingulate cortex, SFG = superior frontal gyrus, STG = superior temporal gyrus.

As for what concerned the association between DMN topology and cognitive flexibility (operationalized by DCCS performance), four of its nodes were observed to present significant linear interactions between their connectivity profile and age. In particular, the left posterior middle temporal gyrus (pMTG) (R^2^ = 0.10, *F*_(16,460)_ = 3.19, *p* < 0.0001) showed a significant interaction between CP and age (*β* = 6.8, *p* = 0.005) and between CC and age (*β* = 5.37, *p* = 0.024), proving that reduced CP and CC are beneficial in the first half of the lifespan (from children to middle age), but not in the older age group (see Figure 7A). Similarly, the left superior frontal gyrus (SFG) (R^2^ = 0.09, *F*_(12,464)_ = 4.09, *p* < 0.0001), left posterior cingulate cortex (PCC) (R^2^ = 0.09, *F*_(12,464)_ = 3.85, *p* < 0.0001) and right precuneus (R^2^ = 0.10, *F*_(16,460)_ = 3.3, *p* < 0.0001) showed a meaningful interaction between their measure of BC and age in association with DCCS performance (left SFG: *β* = −2.36, *p* = 0.014; left PCC: *β* = 1.86, *p* = 0.013, right precuneus: *β* = −2.47, *p* = 0.014), proving a negative association between centrality of the left PCC and performance on the DCCS task in children, with an opposite (positive) association in the older group instead. The opposite trend was instead observed for the left SFG and right precuneus (Figures 7B–D).

Finally, we also observed that the left precuneus (R^2^ = 0.10, *F*_(16,460)_ = 3.28, *p* < 0.0001) and the left STG (R^2^ = 0.9, *F*_(16,460)_ = 2.97, *p* < 0.0001) presented a quadratic relationship between their degree of BC and age in regard of DCCS performance (left precuneus: *β* = 2.85, *p* = 0.002; left STG: *β* = −2.68, *p* = 0.024), with a significant association between BC and DCCS performance that was positive in the younger and the older groups, but negative in the middle-aged group. Similarly, the right precuneus (R^2^ = 0.10, *F*_(16,460)_ = 3.3, *p* < 0.0001) showed a quadratic association between its measures of CP (*β* = 4.26, *p* = 0.039) and CC (*β* = 4.03, *p* = 0.029) with age in respect to DCCS performance, whereby higher CP and CC were positively associated with performance in children and in older adults, but negatively associated in the middle-aged group (Figures 7E,F).

In addition, the left AG (R^2^ = 0.10, *F*_(12,464)_ = 4.34, *p* < 0.0001) and the left pars triangularis (PT) (R^2^ = 0.11, *F*_(12,464)_ = 4.71, *p* < 0.0001) presented significant main effects in their measure of CC (left AG: β = −9.04, *p* = 0.02, left PT: *β* = −8.34, *p* = 0.028) and CP (left AG: *β* = −10.47, *p* = 0.02, left PT: *β* = −11.15, *p* = 0.007) in respect to DCCS performance, regardless of age. Finally, the left paracingulate gyrus (PG) (R^2^ = 0.09, *F*_(12,464)_ = 3.97, *p* < 0.0001) showed a significant association between BC (*β* = −4.26, *p* = 0.048) and DCCS, regardless of age.

A significant main effect of head motion was observed for the left STG (*β* = −18.79, *p* = 0.041), left PG (*β* = −17.95, *p* = 0.046) and left SFG (*β* = −19.19, *p* = 0.032).

**Figure 8:**
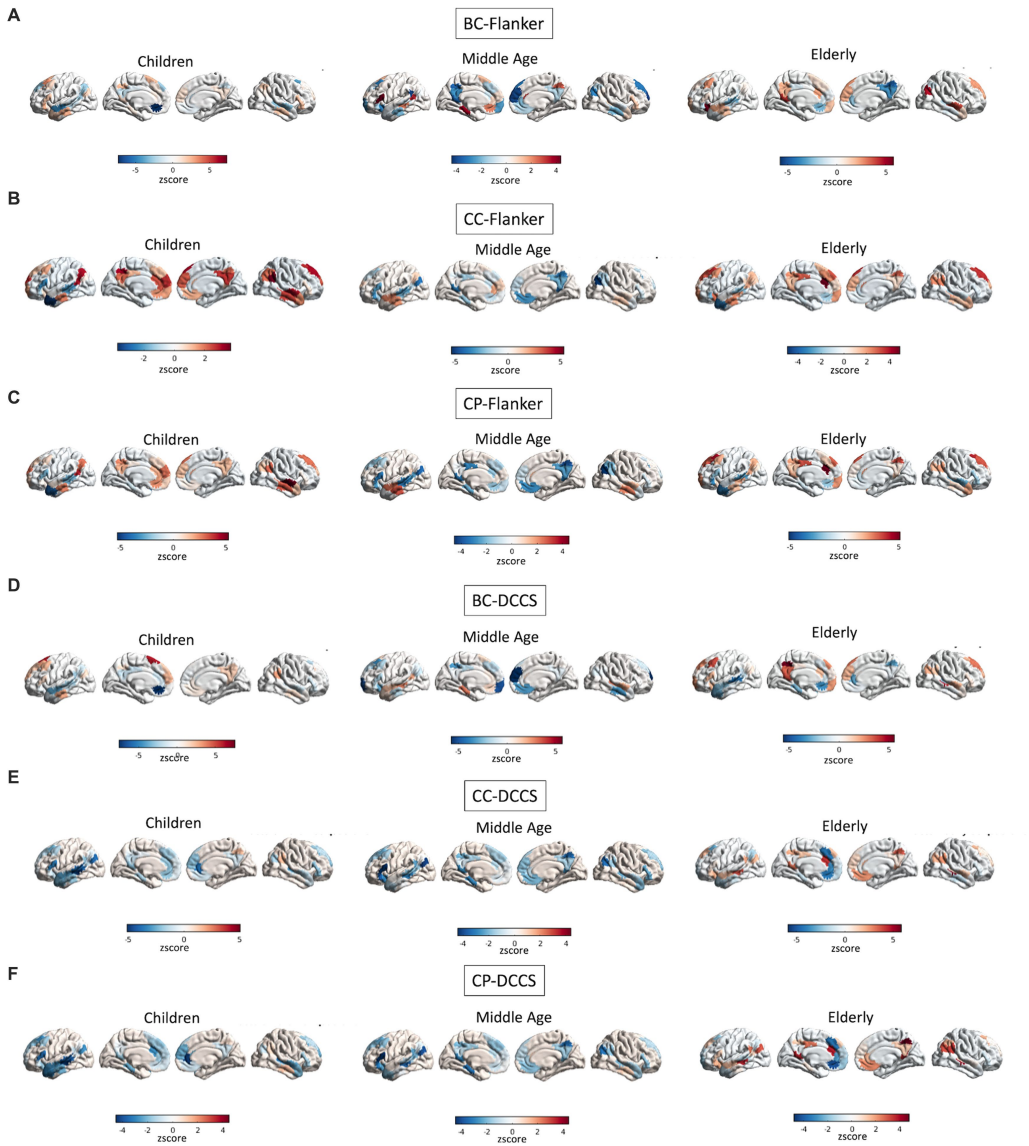
Interactions between age and topology associated with EF performance. Quadratic relationships are shown between topology of all regions of the DMN and performance at the Flanker **(A–C)** and DCCS **(D–F)** tasks. Colors reflect the slope of the interaction in z scores, such as that cooler colors reflect more negative relationships whereas warmer colors reflect more positive relationships.

A full depiction of the relationship between age and topology of the network’s regions associated with EF performance is shown in Figure 8.

### 3.3 Changes in network hubs across the lifespan

**Figure 9:**
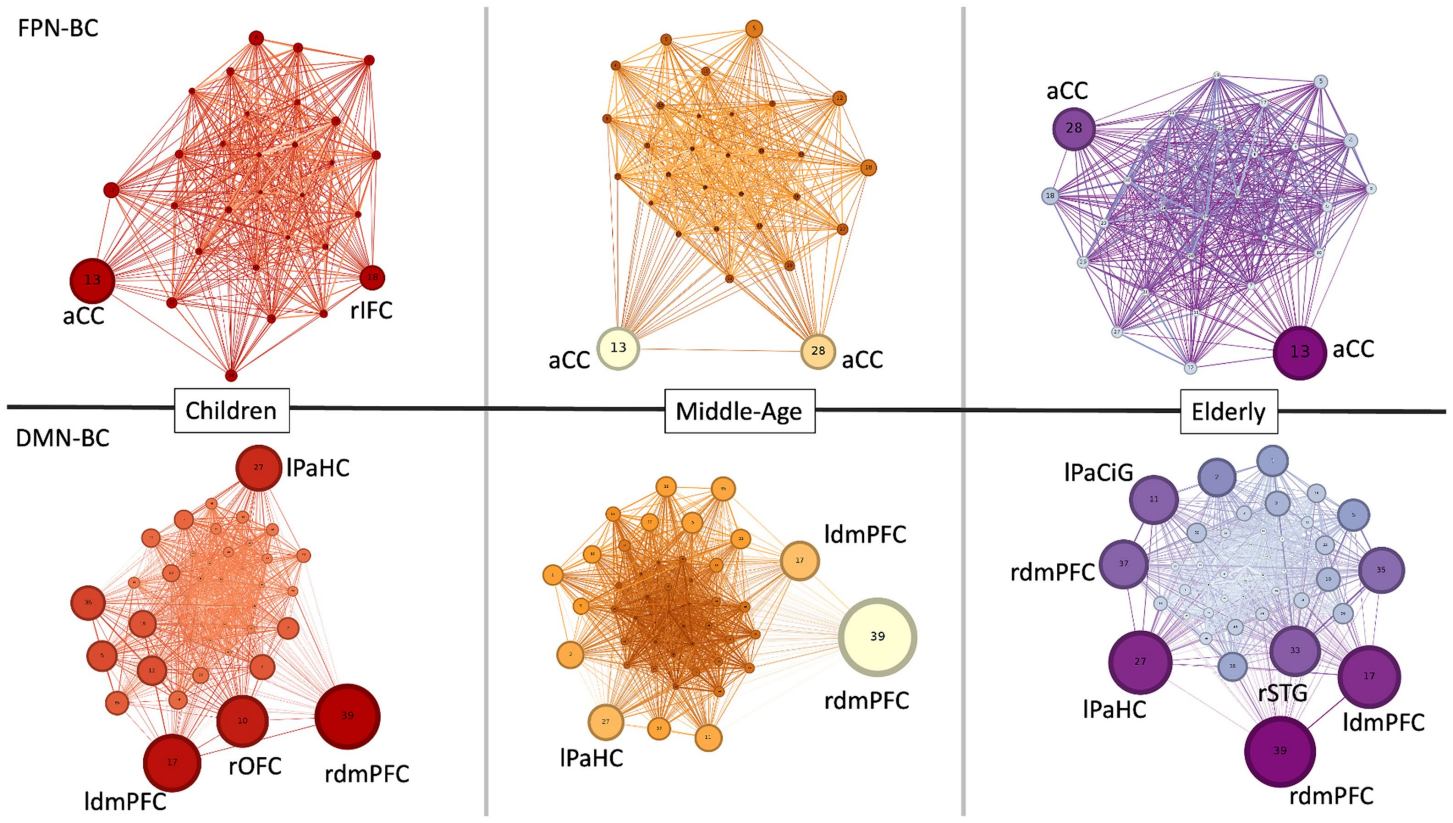
Changes in network hubs across the lifespan. FPN and DMN network hubs are computed based on their measure of BC. For graphic purposes, only the hubs above 1.5 standard deviations are labelled. Nodes’ numbers refer to their parcel number inside the FPN (30 nodes) and DMN (46 nodes), respectively, according to the Schaefer et al. (2018). Size and color of the nodes is proportional to their measure of BC. aCC = anterior cingulate cortex, lPaHC = left parahippocampal cortex, ldmPFC = left dorsomedial prefrontal cortex, lPaCig = left paracingulate gyrus, rIFC = right inferior forntal cortex, rdmPFC = right dorsomedial prefrontal cortex, rSTG = right superior temporal gyrus.

At last, we computed the networks’ hubs by averaging the measures of BC across our young (mean age: 10.4 years), middle-aged (mean age: 47.3 years) and older participants (mean age: 85.7 years) for both the FPN and the DMN. We considered as hubs only the nodes with a measure of BC that was 1.5 standard deviations higher than the group average. We then plotted the obtained results by means of Gephi^3^ (Bastian et al., 2009). We observed that, on average, the distribution of hubs in the FPN tended to remain stable across the lifespan, with a major role of the ACC as a core hub in the network irrespective of age. On the other hand, the DMN showed changes in its number of hubs across the lifespan (Figure 9). In particular, a greater and more diversified number of hubs was present in the younger and older participants, whereas the number of hubs diminished in middle-aged participants. While regions like the bilateral dorsomedial prefrontal cortex (ldmPFC and rdmPFC) and the left parahippocampal cortex (lPaHC) remained stable hubs in the DMN across the lifespan, other regions such as the right orbitofrontal cortex (rOFC), the left paracingulate gyrus (lPaCiG) and the rSTG gain a role as hubs only in children and older participants, suggesting a possible involvement in aiding at the integration of the information at the extremes of the aging curve.

### 3.4 Between-network connectivity of the FPN and DMN

We then tested the association between the average value of participation coefficient for each network (DMN and FPN respectively) and EF measures in both a linear and quadratic fashion. Interestingly, we observed that the average diversity of the connectivity of the FPN towards the DMN was significantly associated with performance on the Flanker task (linear: *r* = 0.13, *p* = 0.004; quadratic: *F*(2,475) = 0.25, *p* = 0.77), but not with performance on the DCCS (linear: *r* = 0.06, *p* = 0.18; quadratic: F(2,475) = 4.25, *p* = 0.015).

As for the DMN, no significant association was observed between its measure of PC and performance on either the Flanker (linear: *r* = 0.08, *p* = 0.08; quadratic: F(2,475) = 1.57, *p* = 0.21) or the DCCS (linear: *r* = 0.03, *p* = 0.46; quadratic: F(2,475) = 0.47, *p* = 0.62) tasks.

## 4 Discussion

The present study sought to investigate the relationship between the intrinsic functional architecture of the brain over time and EF performance. Specifically, it aimed to determine whether normal age-related changes in the topology of the FPN and DMN might provide a neural basis for selective attention and cognitive flexibility abilities across the lifespan. It was hypothesized that within-network topological properties of both the FPN and DMN would change as a function of age, and that this interaction would be significantly associated with behavioral manifestations of both key executive capacities in development and aging. In particular, it was hypothesized that the functional topology of the DMN, a network traditionally conceptualized as suppressed during higher order cognition (for a review see Menon, 2023), would show higher association values with executive capacities across the healthy lifespan than the task-positive FPN, due to its earlier functional maturation and overall role in the integration of information.

To the best of our knowledge, this is the first study directly addressing the role of DMN topological properties in EF performance across the lifespan. Indeed, though neuroimaging studies support the notion that the functional connectome at rest is unique to each individual and that its organizational patterns might be predictive of cognitive performance (Smith et al., 2009), prior studies have either focused on whole brain approaches (Cohen and D’Esposito, 2016; Onoda et al., 2012; Sadiq et al., 2021) rather than on specific functional networks, or have investigated age-related changes on narrower age distributions (Bagarinao et al., 2019; Cohen and D’Esposito, 2016; Stanford et al., 2022) rather than on one spanning from 10 to 100 years. Furthermore, the majority of these studies focused on between-network connectivity, reporting findings similar to the results observed in this study, including an overall decreased functional connectivity strength in aging and a trend towards greater integration, necessary to sustain high-order task execution (Bagarinao et al., 2019; Cohen and D’Esposito, 2016; Onoda et al., 2012; Stanford et al., 2022).

In this study, we observed that topological features of both the FPN and DMN were subject to age-related effects. Indeed, a significant increase in the average CP as a function of age was observed for both networks, suggesting a progressively higher number of steps necessary to link, on average, all within-networks nodes. Because CP represents the inverse of edge weights, such that stronger edges are equivalent to a shorter CP between nodes, this result can also be interpreted from a functional perspective as a progressive weakening of functional correlations. Moreover, an overall reduced segregation characterized both networks as a function of age, as indexed by a reduced CC and MOD for the FPN and a reduced CC for the DMN. In other words, a progressive loss of functional differentiation seems to occur within the FPN with age, favored by an increase in between-module connections, rather than within-module edges, and a decrease in its clusterization. This might result in more distributed information processing at a loss of compartmentalized information. Similarly, the reduced CC observed for the DMN reflects a reduced tendency for nodes to share edges preferentially with nodes that are in spatial proximity (i.e., neighbors), thus also hindering a loss of specialization in favor of a more distributed connectivity pattern with age. An overall significant decrease in GE and SW also characterized the topology of both networks as a function of age, suggesting a progressive loss in network efficiency over the lifespan. Of note, all associations between topology and age were observed to be significantly stronger for the DMN compared to the FPN, consolidating the notion that DMN topology might be more sensitive to age-related changes.

Prior studies have similarly observed a decrease in CP with age at the whole brain level (Bagarinao et al., 2019) and an overall loss of specialization (Edde et al., 2021), while mixed results have been reported in relation to changes in GE as a function of age, for which both positive (Bagarinao et al., 2019) and negative (Stanford et al., 2022) associations have been observed. Again, the apparent contradictions reported in previous research are most likely attributable to the methods employed. Specifically, previous studies mostly considered changes in topology at the whole brain level, and the averaging between several brain regions might have, thus, resulted in the confounding of results. Given that different brain regions are subject to age-related changes to a greater or lesser degree, and that averaging among them leads to the washing out of such variability, a different approach is to consider the networks and their regions individually.

In the second set of analyses, it was of interest to determine whether the topological properties of the FPN and the DMN were associated with EFs, measured with a DCCS and a Flanker task, differently as a function of age. Despite the well-known literature association between FPN and EF performance, we did not observe a significant association between the topology of this network and higher order behavior. Rather, the only significant main effect of the models was that of age, suggesting that performance on the Flanker and DCCS tasks simply improved from childhood to adulthood, without being mediated by FPN topology. Interestingly, these findings might be in line with recent multimodal evidence on properties of the FPN across the lifespan in explaining interindividual differences in EF skills, which proved that grey matter volume, more than resting state-derived topological measures, was a better predictor of performance (Yao et al., 2020). Even so, a significant mediating role for such a measure was observed regarding age-related differences in common EF, but not in shifting-specific and updating-specific EF components (Yao et al., 2020). As our study focused on measures of selective attention and cognitive flexibility, our results appear in line with this prior evidence, leading to the argument that the functional profile of the FPN might fail to show sufficient sensitivity towards such components. Indeed, prior studies looking at the topographical correlates of interindividual differences in EF also reported that shifting and updating-specific abilities were better predicted by variability in the cingulo-opercular, subcortical and ventral attention networks, at least in young healthy individuals (Menardi et al., 2022).

In this study, stronger association between EF performance and topology across the lifespan was observed for the DMN. More specifically, the degree of MOD of the DMN was negatively associated with both Flanker and DCCS performance, suggesting that decreased modularity in the network is associated with better performance on those tasks. This suggests that individuals with higher cognitive flexibility might benefit from more widespread information processing within the DMN. This first result is of particular interest because it suggests that a more widespread distribution of connections within the DMN might explain behavioural flexibility in tasks that require continuous dynamic switching. While this interpretation is only speculative, it aligns with prior literature that has linked the DMN as having a role in average controllability, described as the ability of a node—or a collection of nodes—to steer a system into a variety of activity states (Gu et al., 2015). That is, individuals appear to benefit cognitively from greater integration in the connectivity between DMN nodes, as measured intrinsically during rest. Flexibility at the behavioral level seems, hence, paired with flexibility at the neural level, irrespective of age.

Given that whole-network dynamics might conceal patterns that are specific to single components, however, the same analyses were replicated at a regional level, looking at the association between topology, age and EF. As expected, the topological properties of many single regions forming both the FPN and the DMN were significantly associated with executive performance.

For what concerns the FPN, we first observed that linear relationships between node topology and age were limited to the right hemisphere, whereas more complex, quadratic relationships were limited to the left hemisphere in explaining performance on both the Flanker and the DCCS. Prior work on hemispheric asymmetries of within-network connectivity has highlighted the frontal networks as the most sharply lateralized, with a strong effect of age in shaping homotopic similarities (Agcaoglu et al., 2015). According to the authors, changes in laterality factors with age reflect the cooperative role between homotopic regions in sustaining more complex behaviors (Agcaoglu et al., 2015). Looking at our study, this is interesting as it might suggest a greater sensitivity of the left FPN to age changes. Its quadratic relationship to age implies a supporting role of these regions in EF especially at the extremes of the aging curve. Another interpretation is that the left lateralization of our quadratic effects might reflect its involvement in skills, such as language, that change in a quadratic fashion as a function of age. In support of this, prior studies have also reported that quadratic changes in grey matter volume peak in language cortices and are more prominent in the left than in the right hemisphere (Sowell et al., 2003). However, this interpretation is only speculative. Furthermore, not all language skills fluctuate with age as some that are part of crystallized intelligence (such as reading and semantic representations) remain stable, or even improve, as a function of age (Burke and Shafto, 2011).

For the majority of FPN nodes showing a meaningful interaction between age and topology, it was observed that their increased centrality in the network was positively associated with performance on both the Flanker and the DCCS tasks in older adults. This pattern was true for both linear and quadratic effects of age on this metric. Given the definition of centrality in this study, we can conclude that older adults benefit from a FPN network that is tightly linked, so that communication between its nodes happens rapidly. In particular, higher centrality across several nodes allows transitioning from local information processing to global network communication (Oldham and Fornito, 2019). On the other hand, children seem to benefit from an opposite pattern (i.e., decreased centrality of FPN nodes). As a result, we might argue that the negative association seen between BC and EF performance could reflect the already reported evidence in the literature that children benefit from higher local processing of information in EF tasks, which reflects the progressive emergence of specialized functions (Wang et al., 2019). Interestingly, the relationship between centrality of FPN nodes and performance on the Flanker/DCCS tasks in middle-aged individuals appeared to be less marked, with moderate interactions in between the patterns observed for children and older adults. The only exception is represented by the observed negative interactions between centrality of the left IPL/PCC with Flanker and DCCS performance, respectively, in an opposite fashion to that observed in the younger and older participants. The IPL and the PCC have been associated with interference resolution and filtering of irrelevant information (Berron et al., 2015), as well as monitoring and regulation of neural dynamics (Leech and Smallwood, 2019), such that their increased centrality at the extremes of the aging curve might reflect a greater need for supervision by these regions to ensure task execution. On the contrary, optimization of those processes as a function of maturation, and before neurodegeneration occurs, might require a less central role of these regions to ensure efficient performance.

The main focus of this study, however, was to determine whether the most well-known resting state network, the DMN, could also be meaningfully associated with high order cognition outside of the scanner. As stated above, the modularity of the whole DMN was significantly related to Flanker and DCCS performance, but so were several of its anatomical components when analyzed separately. In particular, we observed that performance on the Flanker task was associated with centrality measures of the bilateral STG, left IFG, left SFG, right AG, and right precuneus. As for what was observed for the FPN, increased centrality of most of these regions was associated with better performance on the Flanker task in older adults, but not in children or middle-aged individuals. The opposite trend was instead observed for the left IFG, left SFG and the right precuneus, whose increased centrality exerted a negative effect on elderlies’ performance. Interestingly, such key hubs in the DMN have been reported to play an active role in Flanker performance during task fMRI, such that their activation was most prominent when the individual was required to inhibit an inappropriate response, namely to be most active when response to a stimulus was not required (Anderson et al., 2016). This evidence favors the conception of DMN activity as related to the preparedness of the individual to respond to a stimulus more in general (Anderson et al., 2016), which again falls with the suggested role of the DMN to mediate the fast transitioning toward a multitude of states (Gu et al., 2015). This aligns with the interpretation that the centrality of DMN hubs could mediate its ability to integrate information (Buckner et al., 2009) and help in modulating the promptness of this system to disengage from a resting state condition and allow task-relevant activity to occur, or the opposite, hence mediating performance outside the scanner. As DMN undergoes continuous progressive increased connectivity in its posterior-to-anterior axis during childhood (Rebello et al., 2018; Ricardo Sato et al., 2014), followed by decreased connectivity in later age (Vidal-Piñeiro et al., 2014), it is not surprising that those older adults showing the highest centrality of these hubs, indicative of more preserved connectivity, are the ones who perform better. Furthermore, given the reported role of these regions in contextual information processing (Ames et al., 2015; Anderson et al., 2016), we argue that the centrality of DMN nodes might influence how the information from the environment is used by the individual. In particular, the centrality of these regions might modulate the amount of information coming from the environment that is used by the individual to aid in the execution of the task. The interaction that we observed with age suggests that high centrality is beneficial in the older individuals, such that they might rely more on contextual information to direct their attention. On the other hand, this might represent a source of noise in children, such that the excessive centrality of these regions and the related amount of contextual information that is processed could be detrimental to their performance. An alternative hypothesis is that the preferred reduced centrality in children might simply be the result of diminished connectivity between DMN nodes, typical of this age group (Rebello et al., 2018; Ricardo Sato et al., 2014). This would be in line with the observed negative correlation between DMN connectivity strength and DCCS performance (see Supplementary Figure S5), for which lower connectivity values, driving the negativity of the correlation, are observed in the younger counterpart of the sample.

Aside from centrality measures, we also observed that increased clusterization and path length of the right AG and right precuneus were also positively correlated with Flanker performance in children and older adults, but negatively in middle-aged individuals. Both measures suggest a favorable impact of local information processing on performance of children and older adults, but not in middle-aged individuals. The higher local processing seen in children can be interpreted with the “local to distributed” organization principles (Fair et al., 2009), which suggests that communities of nodes in children emerge from simple anatomical proximity of regions, which will then evolve in a functionally distributed information processing as a result of brain maturation and experience (Fair et al., 2009). As the brain ages further, the distributed information processing progresses to the extreme, resulting in the loss of brain segregation in aging, which has been found predictive of long-term cognitive functioning in the older adults (Chan et al., 2014). In this study, older adults with increased local processing (higher CC and CP) in the right AG and the right cuneus were observed to have higher performance at the Flanker task, which might suggest they displayed neural patterns closer to a younger counterpart, resulting in more favorable cognitive performance.

Notably, similar topological patterns were observed in association with DCCS performance as well, with an even greater number of DMN nodes significantly associated with cognitive flexibility as a function of age: the left pMTG, left SFG, left PCC, left STG and the bilateral precuneus. Compared to the FPN, the DMN seemed to show a left predominance of the regions whose topological properties showed meaningful interactions with age and DCCS performance. These results appear in line with prior studies from the literature suggesting a left lateralization of within-network connectivity of the DMN (Agcaoglu et al., 2015) and of cognitive flexibility (Kim et al., 2012; Yin et al., 2015). In particular, criterion setting, central in DCCS tasks, has been associated with left PFC involvement (Vallesi, 2012, 2021), which embeds the left SFG reported in this study. As for what concerns the other regions in the DMN that emerged as significant, namely the left pMTG and the left PCC, both have been reported to cooperate in ensuring optimal execution in high demanding cognitive control tasks (Cocchi et al., 2013; Davey et al., 2016). Furthermore, given their role in the integration of multisensory information and in the coordination of activity among specialized systems (Cocchi et al., 2013; Davey et al., 2016), their role in high order behavior has been extensively reported, such as in creative thinking (Beaty et al., 2015; Davey et al., 2016; Fink et al., 2010; Leech and Smallwood, 2019).

Overall, the functional activity of a widespread set of regions in the inferior frontal junction and the posterior parietal cortex has been reliably found as part of a meta-analytically derived set of regions involved in switching-related tasks (Kim et al., 2012). We provide support for these prior studies by highlighting how such switch-related meta-regions, carefully reported based on an extensive review of task-evoked fMRI studies (Kim et al., 2012), can in fact be found even when looking solely at their connectivity profiles at rest.

Lastly, we observed that an easier communication between the left pMTG to the rest of the DMN (lower CP) ensured better performance in children and middle-aged adults, with minimum positive effect on the older adults instead. These results mirror the functional refinement reported to occur across the lifespan, with a progressive increase in integration measures until the age of 40 (Edde et al., 2021). In contrast to what was observed for the Flanker task, for which the centrality of DMN nodes was a critical factor, here its impact appeared modest but with the same trends; that is, with a particular positive effect on cognitive flexibility performance in the older adults.

Based on the observed patterns, we can conclude that EF performance across the lifespan seems to rely on local information processing in children, reflective of the emergence of specialization with the brain network. As the individual matures, EF performance starts to depend more on the integration between networks’ nodes, possibly reflective of a more efficient parallel processing of the information. As the brain ages further, the distributed information processing progresses to the extreme, resulting in the loss of brain segregation in aging and the possible presence of compensatory mechanisms. Of interest, those topological changes seem to be more marked in the DMN, which might explain its tighter link to individual performance.

We wish to conclude by discussing two additional results in our analyses. First, despite the extensive number of studies that have investigated the FPN-DMN interplay in explaining high cognitive functioning (Cocchi et al., 2013; Hearne et al., 2015, 2016; Menon and D’Esposito, 2021; Shi et al., 2018), we decided to test the association between the diversity of their intermodular connections with EF performance. We hence observed that the higher the average amount of FPN-DMN connections, rather than FPN-FPN connections, the better the performance on the Flanker task. This appears in line with the suggested “gate-keeping role” of the FPN in mediating goal-directed cognition by orchestrating the dynamic interplay between other resting state networks, such as the DMN (Spreng et al., 2013). In particular, the participatory role of FPN nodes towards the DMN appears beneficial in selective attention, probably by favoring the integration of information between the two networks (Spreng et al., 2013).

Second, head motion often appeared as a significant main factor in our analyses, suggesting that individuals who moved less inside the scanner had a better cognitive performance as assessed by the Flanker and DCCS tasks. Despite the very low level of movement in the sample (mean = 0.2 mm, std. = 0.09 mm), these results replicate previous findings (Bolton et al., 2020; Hausman et al., 2022) on how even small head movements (in putatively clean sets of timepoints) carry behaviorally relevant information. Nevertheless, none of the interactions between our graph theory measures and head movement were significantly associated with task performance, suggesting that motion exerts a stand-alone effect in the analyses.

## 5 Limitations and future directions

While our study addressed an important gap in the literature by focusing on within-network functional connectivity of the DMN in association with EF abilities, a few limitations should be noted. First, regions of the FPN and DMN were defined with the Schaefer et al. (2018). However, a general consensus is lacking in terms of which brain parcellation system is optimal for brain network construction in both adults and children (Han et al., 2018). Because the final topological properties of a network are ultimately biased by the method chosen, future studies might consider employing different parcellation schemes to determine if the present findings are replicable. For instance, prior studies found meaningful associations between interindividual differences in shifting-specific abilities and the cingulo-opercular network, instead of the FPN, which is represented in the parcellation of Power et al. (2011) but not in that of Schaefer et al. (2018). Because we did not investigate the association between topology, age and EF in other resting state networks outside of the FPN and DMN, we cannot exclude that other networks might show significant associations with high order behavior. However, this aspect has already been covered by several studies in the literature that addressed between-network connectivity changes in respect to EF, whereas none selectively looked at the roles of the FPN and DMN as in this study.

In this regard, it has been reported that the extent of modular reorganization with age can cause the DMN and FPN to be identified as a single module in the older population (Geerligs et al., 2015). This and other findings on the intra- and inter-individual variability of the functional connectome (Laumann et al., 2015) might lead to questioning the appropriateness of employing group-derived atlases in lifespan studies, since these parcellations are usually derived using samples of young adults between 18 and 35 years of age. A potential solution stands in the use of individual-specific parcellations, allowing ROIs boundaries to change across individuals to better reflect their true functional and structural correspondence (Chong et al., 2017; Kong et al., 2019). Despite the fact that those approaches are proving to be highly advantageous, to the best of our knowledge, no study has yet attempted to apply this methodology to the parcellation of much younger or older brains, where the interindividual variability in functional connectivity may be even greater. As such, even recent work on normative models depicting lifespan trajectory changes in functional connectivity has necessarily relied on the use of group-level parcellations (Rutherford et al., 2023). Future studies are needed to develop reliable cortical parcellations across the lifespan.

Furthermore, HCP Lifespan data have been preprocessed based on the existing HCP preprocessing pipelines, which involve normalization of the volumetric data by means of nonlinear coregistration to the MNI template, while the surfaces are mapped to the standard fs_LR_32k space using spherical registration and surface downsampling (Glasser et al., 2013). This might cause a bias in the greater amount of warping necessary to bring very young/old brains in the space of a common template built from a population of young adults. The process of normalizing the data to a common template in lifespan studies represents indeed a technical challenge when trying to harmonize data across age groups (Harms et al., 2018). On the positive side, cortical surface registration that relies on areal features, used in the HCP multimodal pipeline for data alignment, is far superior to traditional volume-based coregistrations (Coalson et al., 2018) and might therefore help improve spatial localization across the lifespan. It has also been reported that brain volume reaches 90% of the adult brain size by the age of 6 (Stiles and Jernigan, 2010) and the head size of children >7 years of age is sufficiently similar to that of adults to allow the same head coil to be used for MRI data acquisition (Harms et al., 2018). On the other hand, a smaller pediatric coil was used for the acquisition of data in 5–7 years old participants (Harms et al., 2018). As the present study only analyzed data of individuals ≥ 10 years of age, we believe that the risk of bias in structural and functional normalization across age groups was reduced as much as possible, albeit still not being as optimal as using age-specific templates.

While several common frameworks of the EF system assume that its core components are working memory, selective attention, and cognitive flexibility (Diamond, 2012; Miyake et al., 2000), the present study considered only selective attention and cognitive flexibility. Given that working memory is intertwined in the ability to select attention and to shift focus, it would be critical to consider how working memory performance alone might be associated with the functional topology of the DMN. It is also worth mentioning that the models’ accuracy in explaining EF performance in this study emerged as moderate, in line with the known small effect size typical of brain-behavior associations studies (Marek et al., 2022). Aside from the issue of sample size, granularity mismatch between neuroimaging and cognitive measures, as well as insufficient phenotypic complexity/resolution and measurement non variance in cognitive testing can limit even further the magnitude of brain-behavior associations (Tiego and Fornito, 2022). As such, future studies should consider a more in-depth cognitive evaluation to overcome the lack of sufficiently complex and exhaustive measures of behaviour that often characterize large sample studies.

Our study made use of data coming from the HCP Lifespan database, which merges the Developmental and the Aging projects (Harms et al., 2018). As such, it does not include participants between 21 to 36 years of age, which were extensively studied in the HCP Young Adult dataset (Van Essen et al., 2013).

Lastly, our study relies on a cross-sectional design investigation, which can be considered suboptimal compared to longitudinal approaches. As such, we are limited on the type of inferences we can draw from our results, which remain highly correlational and not causal in nature. Longitudinal scan acquisitions are now being performed for some big cohorts (e.g., UK Biobank) and will hopefully help bridge the gap in brain-behavior association studies across the lifespan in the future.

## 6 Conclusion

The results of this study point to a promising resting state neural basis of the EF system. In particular, we demonstrate that the topological organization of both the FPN and the DMN are associated with high order behavior outside of the scanner, and suggest a tight link between neuro-functional and cognitive-behavioral efficiency. Interestingly, our results also suggest that DMN topology might be more sensitive to age-related changes, as well as more sensitive to a hemispheric specificity of cognitive flexibility, as evinced from the reported left-hemisphere dominance of regions whose topology meaningfully interacted with age and DCCS performance. Similarly, both the topology of the whole DMN, as well as that of its single components, were associated with EF performance, whereas the topological properties of only single regions within the FPN were related to EF. Because the DMN is present and matures earlier in life than the FPN, and because it can be more easily measured in otherwise challenging populations (e.g., pediatric individuals, older adults, or neurologically impaired patients), we argue that the study of its topology in association with higher order cognition across the lifespan might be of greater interest compared with other resting state networks.

## Data Availability

Data and/or research tools used in the preparation of this manuscript were obtained from the National Institute of Mental Health (NIMH) Data Archive (NDA). NDA is a collaborative informatics system created by the National Institutes of Health to provide a national resource to support and accelerate research in mental health. Dataset identifier(s): [10.15154/pvjz-h270].

## Footnotes

^1^ https://www.humanconnectome.org/lifespan-studies

^2^ https://sites.google.com/site/bctnet/

^3^ https://gephi.org/
