# Supplementary material for: Brain topology underlying executive functions across the lifespan: focus on the default mode network
Source: Front Psychol. 2024 Sep 4;15:1441584. doi: 10.3389/fpsyg.2024.1441584 (PMC11408365; doi:10.3389/fpsyg.2024.1441584)
Supplement: Supplementary file 1 [file Data_Sheet_1.DOCX]

**Supplementary Material**

1. **Association between EF measures and graph theory properties of a control network**

We re-run our analyses on a control network, the sensorimotor network (SMN), whose connectivity properties are not expected to correlate with executive functions. The overall multiple regression models, which considered age, gender, head motion and SMN topology, as well as their interaction in relation to Flanker and DCCS performance emerged as significant (Flanker: R^2^ = 0.07, *F*_(12, 459)_ = 2.91, *p* = 0.0006; DCCS: (R^2^ = 0.07, *F*_(12, 465)_ = 3.27, *p* = 0.0001). However, the only significant association with performance in the models was the linear effect of age (Flanker: β = 3.15, *p* < 0.0001, DCCS: β = 4.03, *p* = 0.0001) and motion (Flanker: β = -16.95, *p* = 0.008; DCCS: β = -22.02, *p* = 0.01), suggesting that variability in the performance scores can be related to differences in age rather than topological properties of the SMN. Furthermore, it suggests that individuals with less head motion are the ones who perform better at the cognitive batteries administered outside the scanner. Importantly, no significant interaction between our graph theory measures and head motion was observed.

1. **FPN Network-level analyses**

The overall multiple regression models, which considered age, gender, head motion and FPN topology, as well as their interaction in relation to Flanker and DCCS performance emerged as significant (Flanker: R^2^ = 0.08, *F*_(12, 459)_ = 3.36, *p* = 0.0001; DCCS: R^2^ = 0.08, *F*_(12, 464)_ = 3.31, *p* = 0.0001). For both models, the linear effect of age was observed to be meaningfully associated with performance on the EF tasks (Flanker: β = 2.66, *p* = 0.0001, DCCS: β = 5.13, *p* < 0.0001). Head motion was also associated with Flanker performance (β = -16.57, *p* = 0.011), suggesting that those individuals moving the most inside the scanner were also the ones with the worst cognitive performance on the task.

These results replicate what was already observed with the 200 Parcels Schaefer’s Atlas.

- 1. **FPN Node-level analyses**

The right inferior frontal gyrus (IFG) (R^2^ =0.08, *F*_(12,459)_ = 3.72, *p* < 0.0001) and the right posterior cingulate cortex (PCC) (R^2^ = 0.09, *F*_(12,459)_ = 3.78, *p* < 0.0001) showed a significant interaction between CP and age with respect to Flanker performance (IFG: β = -2.57, *p* = 0.013; PCC: β = 3.07, *p* = 0.033). The interaction between age and CC was associated to Flanker performance for the right PCC (β = 3.06, *p* = 0.016) and the right precuneus (R^2^ =0.09, *F*_(12,459)_ = 3.64, *p* < 0.0001, β = 2.99, *p* = 0.032). For the left anterior cingulate gyrus (aCG), the interaction between BC and age was significantly associated with performance on the Flanker task (R^2^ =0.09, *F*_(12,459)_ = 3.76, *p* < 0.0001, β = -1.61, *p* = 0.017).

In a quadratic fashion, the left middle temporal gyrus (MTG) (R^2^ = 0.10, *F*_(16,455)_ = 3.2, *p* < 0.0001) and the right frontal pole (FP) (R^2^ = 0.09, *F*_(16,455)_ = 2.93, *p* = 0.0001) showed a significant association between the interaction of age with CP (MTG: β = 3.22, *p* = 0.021; FP: β = 4.37, *p* = 0.003) and CC (MTG: β = 3.16, *p* = 0.021; FP: β = 3.65, *p* = 0.01) with respect to Flanker performance.

Finally, the left posterior supramarginal gyrus (pSMG)(R^2^ = 0.09, *F*_(12,459)_ = 3.65, *p* < 0.0001), the left anterior dorsolateral prefrontal cortex (aDLPFC) (R^2^ = 0.08, *F*_(12,459)_ = 3.37, *p* < 0.0001) and the right aDLPFC (R^2^ = 0.08, *F*_(12,459)_ = 3.49, *p* < 0.0001) showed significant interaction between their measures of CP (pSMG: β = 8.86, *p* = 0.028; left aDLPFC: β = 9.15, *p* = 0.016) and CC (pSMG: β = 8.74, *p* = 0.017; left aDLPFC: β = 8.9, *p* = 0.01;right aDLPFC: β = 7.78, *p* = 0.026) in association with Flanker performance, unrelated to age.

For many nodes, motion emerged as a significant main effect in association to Flanker performance (right IFG: β = -18.13, *p* = 0.004; right PCC: β = -21.52, *p* = 0.001; right precuneus: β = -18.72, *p* = 0.003; left aCG: β = -16.59, *p* = 0.009; left MTG: β = -20.4, *p* = 0.001, right FP: β = -15.82, *p* = 0.016, left pSMG: β = -18.74, *p* = 0.004, left aDLPFC: β = -18.19, *p* = 0.005, right aDLPFC: β = -20.19, *p* = 0.001).

Overall, these results replicate the involvement of the SMG, precuneus and PCC already observed with the 200 parcels of the Schaefer Atlas (see main manuscript), but further highlighted the contribution of additional ROIs in the frontal, temporal and cingulate cortex (see Figure S1).


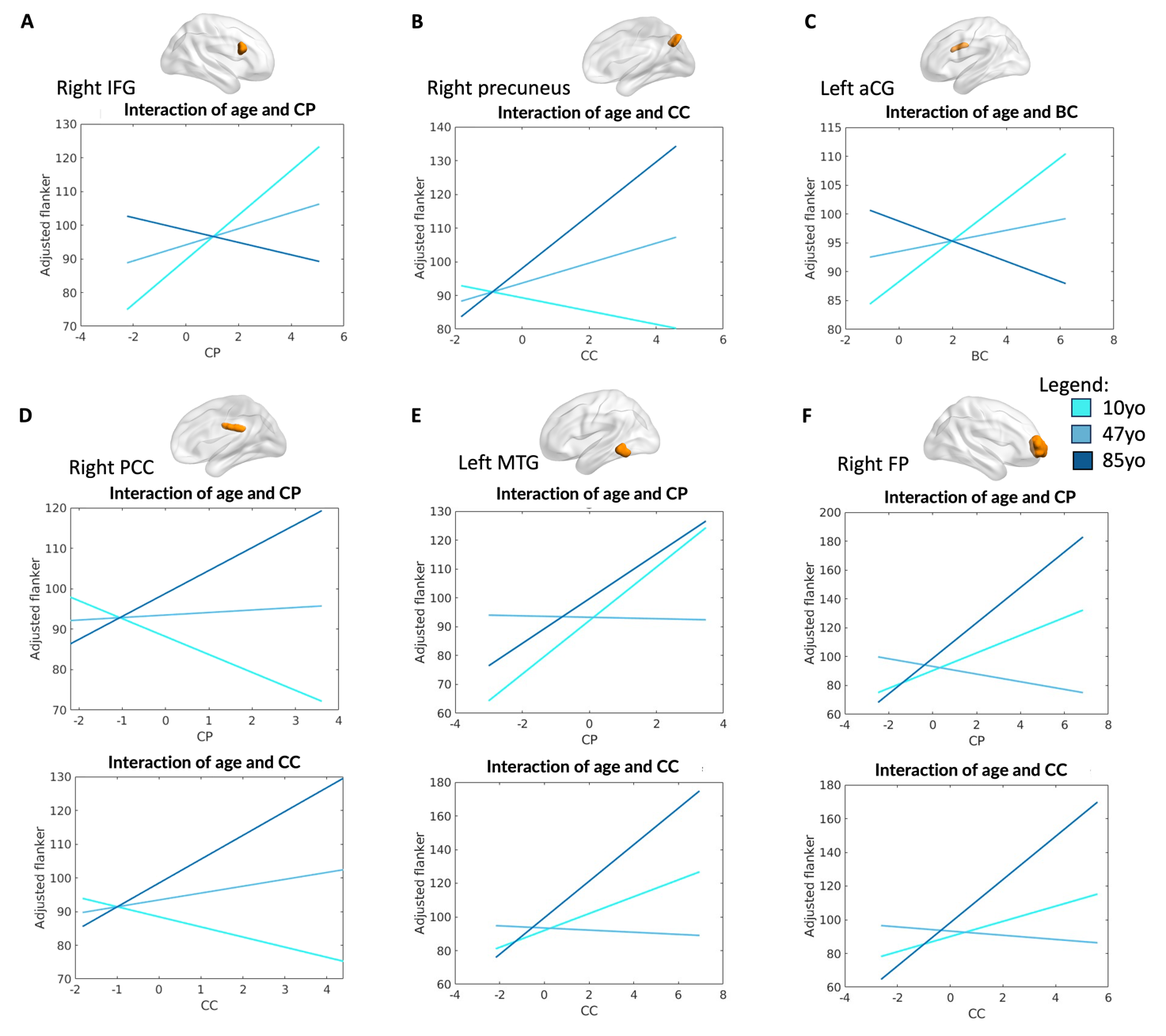


***Figure S1*. Linear and quadratic interactions between FPN topology and age in Flanker performance.** Significant linear interactions between age and graph theory measures in relation to Flanker performance are presented graphically for the right IFG (**A**), right precuneus (**B**), left aCG (**C**) and right PCC (**D**). On the other hand, significant quadratic interactions between age and graph theory measures in relation to Flanker performance were observed for the left MTG (**E**) and the right FP (**F**). Age is subdivided into three categories for interpretability, with the average age of each group shown: 10 yo = 10 years old; 48 yo = 48 years old; 85 yo = 85 years old. aCG = anterior cingulate gyrus, BC = betweenness centrality, CC = clustering coefficient, CP = characteristic path length, IFG = inferior frontal gyrus, FP = frontal pole, MTG = middle temporal gyrus, PCC = posterior cingulate cortex.

For what concerns performance on the DCCS, three regions showed a significant interaction between BC and age with respect to task performance: the left pSMG (R^2^ = 0.08, *F*_(12,464)_ = 3.73, *p* < 0.0001; β = 1.69, *p* = 0.027), the left aCG (R^2^ = 0.08, *F*_(12,464)_ = 3.58, *p* < 0.0001; β = -2.04, *p* = 0.031) and the right superior frontal gyrus (SFG) R^2^ = 0.08, *F*_(12,464)_ = 3.27, *p* < 0.0001; β = 1.92, *p* = 0.049).

All three regions also showed a main effect of head motion (left pSMG: β = -17.8, *p* = 0.047; left aCG: β = -19.53, *p* = 0.029; right SFG: β = -21.07, *p* = 0.014).

Overall, the results replicate the role of the right SFG in association to DCCS performance (see main manuscript), but also highlight the contribution of two additional ROIs in the cingulum and parietal cortex (see Figure S2).


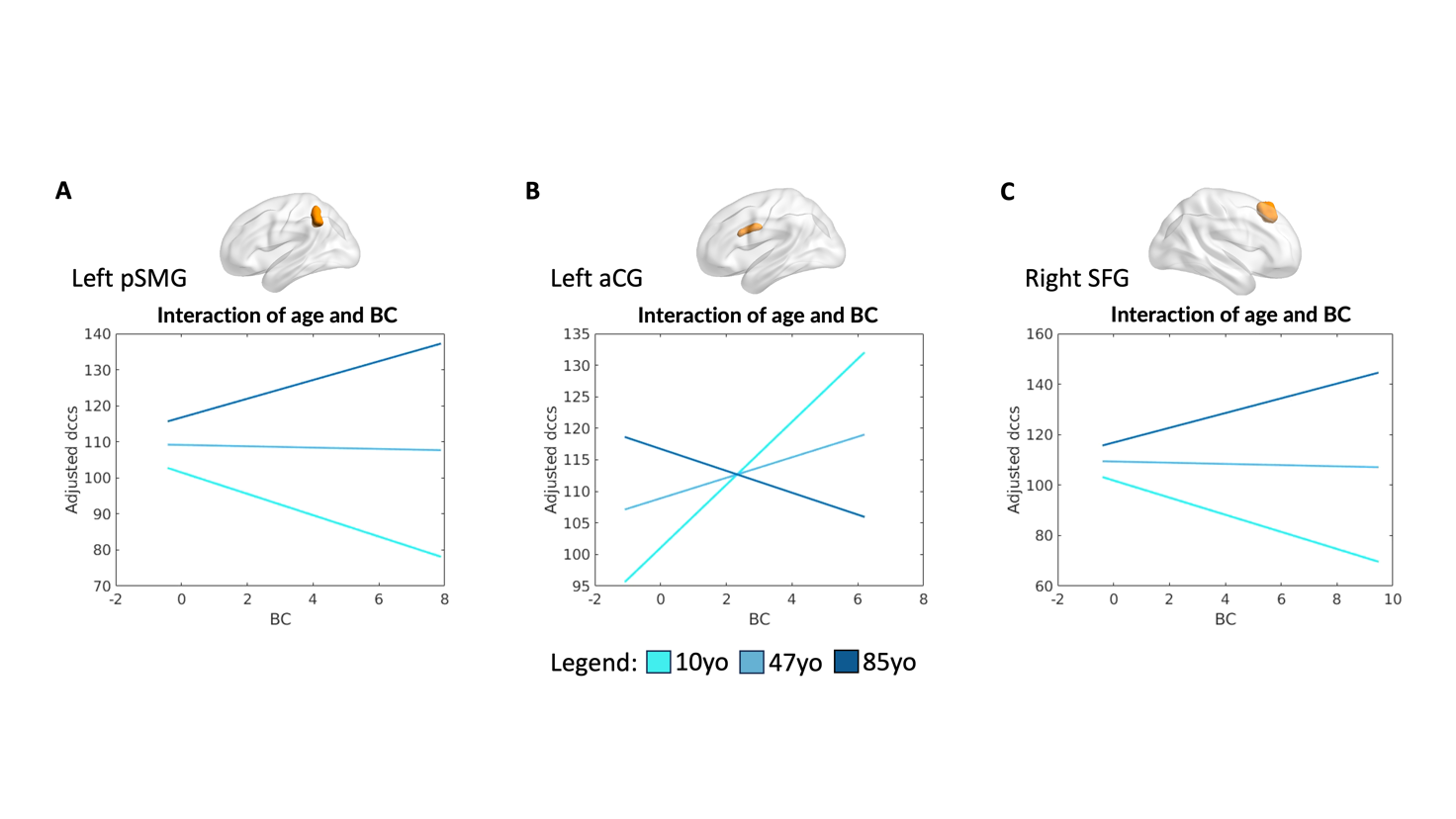


***Figure S2*. Linear interactions between FPN topology and age in DCCS performance.** Significant linear interactions between age and graph theory measures in relation to DCCS performance are presented graphically for the left pSMG (**A**), left aCG (**B**), and right SFG (**C**). Age is subdivided into three categories for interpretability, with the average age of each group shown: 10 yo = 10 years old; 48 yo = 48 years old; 85 yo = 85 years old. aCG = anterior cingulate gyrus, BC = betweenness centrality, pSMG = posterior supramarginal gyrus, SFG = superior frontal gyrus.

1. **DMN- Network-level analyses**

The overall multiple regression models, which considered age, gender, head motion and DMN topology, as well as their interaction in relation to Flanker and DCCS performance emerged as significant (Flanker: R^2^ = 0.10, *F*_(12,459)_ = 4.45, *p*< 0.0001; DCCS: (R^2^ = 0.08, *F*_(12, 464)_ = 3.66, *p* < 0.0001). For both tasks, we observed a main effect of MOD (Flanker: β = -6.42, *p* = 0.0002; DCCS: β = -5.41, *p* = 0.029). In addition, age was associated with DCCS performance (β = 3.91, *p* = 0.0006), as did head motion with respect to Flanker performance (β = -15.93, *p* = 0.018), suggesting that those moving the most inside the scanner were also the ones with the worst cognitive performance on the task.

These results replicate what was already observed with the 200 parcel Schaefer’s Atlas (see main manuscript).

- 1. **DMN- Node-level analyses**

The right posterior MTG (R^2^ =0.07, *F*_(12,459)_ = 2.96, *p* = 0.0005) and the left SFG (R^2^ = 0.09, *F*_(12,459)_ = 2.85, *p* < 0.0001) both showed a significant interaction between BC and age in respect to Flanker performance. The effect was observed to be linear for the right pMTG (β = 1.59, *p* = 0.044) and quadratic for the left SFG (β = 1.87, *p* = 0.015). A series of other regions showed meaningful association between their topology and Flanker performance, although without an interaction effect with age: the right middle frontal gyrus (MFG) showed an effect of BC (R^2^ =0.07, *F*_(12,459)_ = 3.05, *p* < 0.0001; β = -2.86, *p* = 0.043), the right SFG showed an effect of CP (R^2^ = 0.09, *F*_(12,459)_ = 3.79, *p* < 0.0001; β = 7.73, *p* = 0.015), and both the left (R^2^ = 0.07, *F*_(12,459)_ = 2.99, *p* = 0.0005) and right (R^2^ = 0.08, *F*_(12,459)_ = 3.42, *p* < 0.0001) precuneus showed an effect of CC (left: β = 6.81, *p* = 0.032; right: β = 6.98, *p* = 0.027).

All regions also showed a significant main effect of head motion in association to Flanker performance (right pMTG: β = -20.19, *p* = 0.001; left SFG: β =-17.62 , *p* = 0.007; right MFG: β = -18.20, *p* = 0.005; right SFG: β = -17.38, *p* = 0.007;left precuneus: β = -16.81, *p* = 0.012; right precuneus: β = -17.97, *p* = 0.006).

In comparison to the results reported in the main manuscript, the left SFG showed the same effect for the younger and middle age groups, but an opposite trend for the elder population. Additional results emerged for the right pMTG, which showed the same effect as what reported for the STG in the main manuscript (see Figure S3).

**
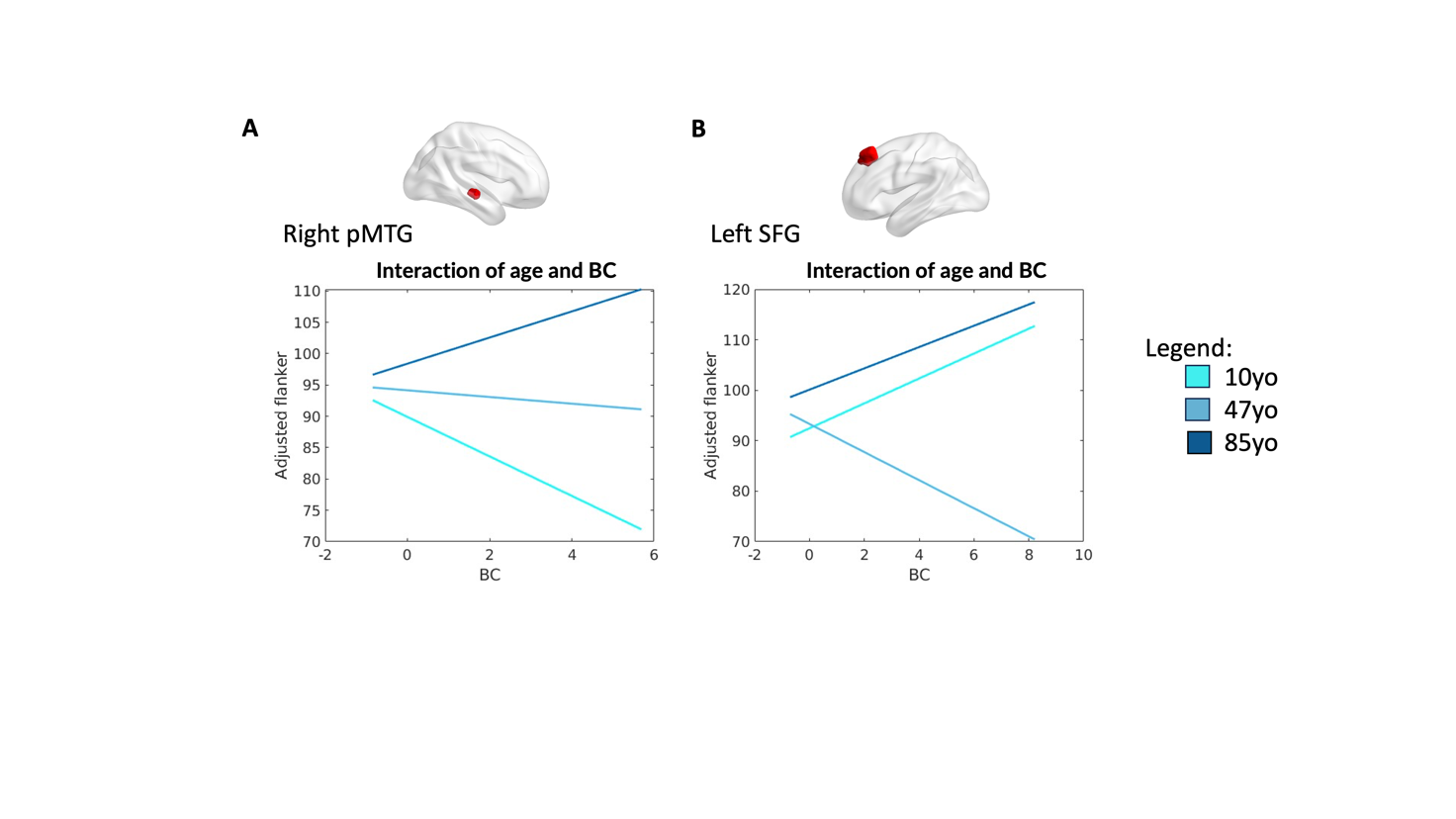
**

***Figure S3*. Linear and quadratic interactions between DMN topology and age in Flanker performance.** Significant interactions between age and BC in association to Flanker performance are presented graphically for the right pMTG (**A**) and left SFG (**B**). Age is subdivided into three categories for interpretability, with the average age of each group shown: 10 yo = 10 years old; 48 yo = 48 years old; 85 yo = 85 years old. BC = betweenness centrality, pMTG = posterior middle temporal gyrus, SFG = superior frontal gyrus.

The left precuneus (R^2^ = 0.08, *F*_(12,464)_ = 3.55, *p* < 0.0001) and the right posterior MTG (R^2^ = 0.09, *F*_(12,464)_ = 2.99, *p* < 0.0001) showed a significant interaction between CP and age in association to the DCCS task (left precuneus: β = 4.16, *p* = 0.037; right pMTG: β = 5.43, *p* = 0.027). The right pMTG also showed a significant quadratic interaction between CC and age (β = 4.58, *p* = 0.012). On the other hand, the interaction between BC and age was associated to DCCS performance linearly for the right anterior ventral PFC (R^2^ = 0.10, *F*_(12,464)_ = 4.34, *p* < 0.0001; β = 2.08, *p* = 0.026) and in quadratic fashion for the left superior parietal lobule (SPL) (R^2^ = 0.11, *F*_(12,464)_ = 3.43, *p* < 0.0001; β = 2.93, *p* = 0.005).

A main effect of motion was observed for the left precuneus (β = -18.92, *p* = 0.041) and the right pMTG (β = -20.15, *p* = 0.026).

In addition, a parcel in the posterior portion of the SPL (R^2^ = 0.08, *F*_(12,464)_ = 3.63, *p* < 0.0001), the left pars triangularis (PT) (R^2^ = 0.11, *F*_(12,464)_ = 4.7, *p* < 0.0001) and the left pars opercularis (R^2^ = 0.09, *F*_(12,464)_ = 4.05, *p* < 0.0001) showed a significant association between CP (pSPL: β = -9.75, *p* = 0.038; PT: β = -10.55, *p* = 0.017; PO: β = -9.78, *p* = 0.026) and CC (pSPL: β = -8.32, *p* = 0.044; PT: β = -8.58, *p* = 0.018) with DCCS performance, regardless of age.

These results replicate the role of the pMTG in association to DCCS performance (see main manuscript) and provide additional evidence on the role of regions located in the posterior parietal cortex and ventral prefrontal cortex (see Figure S4).


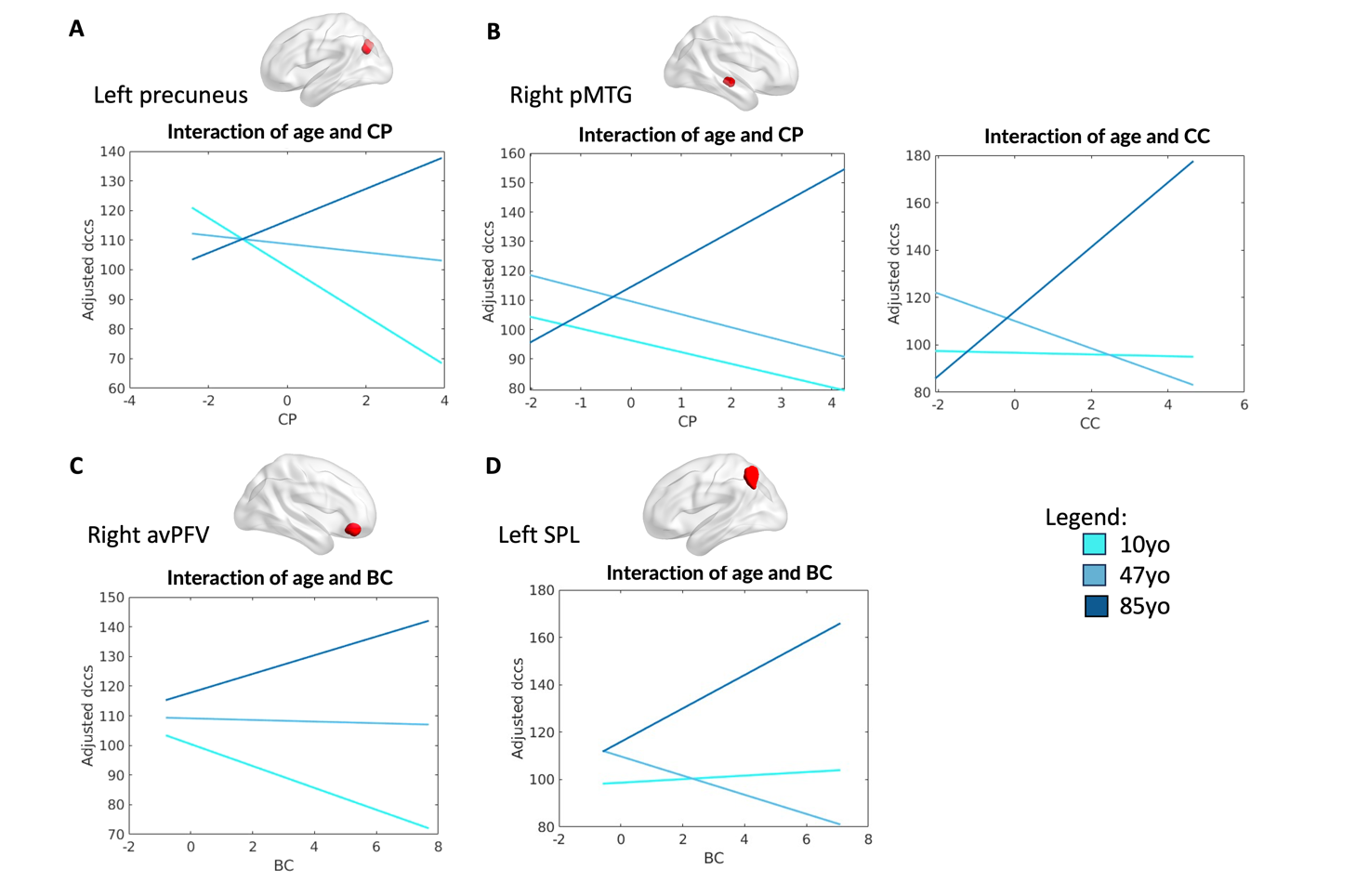


***Figure S4*. Linear and quadratic interactions between DMN topology and age in DCCS performance.** Significant interactions between age and graph theory measures in relation to DCCS performance are presented graphically for the left precuneus (**A**), right pMTG (**B**), right avPFC (**C**) and left SPL (**D**). Age is subdivided into three categories for interpretability, with the average age of each group shown: 10 yo = 10 years old; 48 yo = 48 years old; 85 yo = 85 years old. avPFC = anterior ventral prefrontal cortex, BC = betweenness centrality, CC: clustering coefficient; CP = characteristic path length; pMTG = posterior middle temporal gyrus; SPL = superior parietal lobule.

1. **Within-network connectivity strength and EF performance**


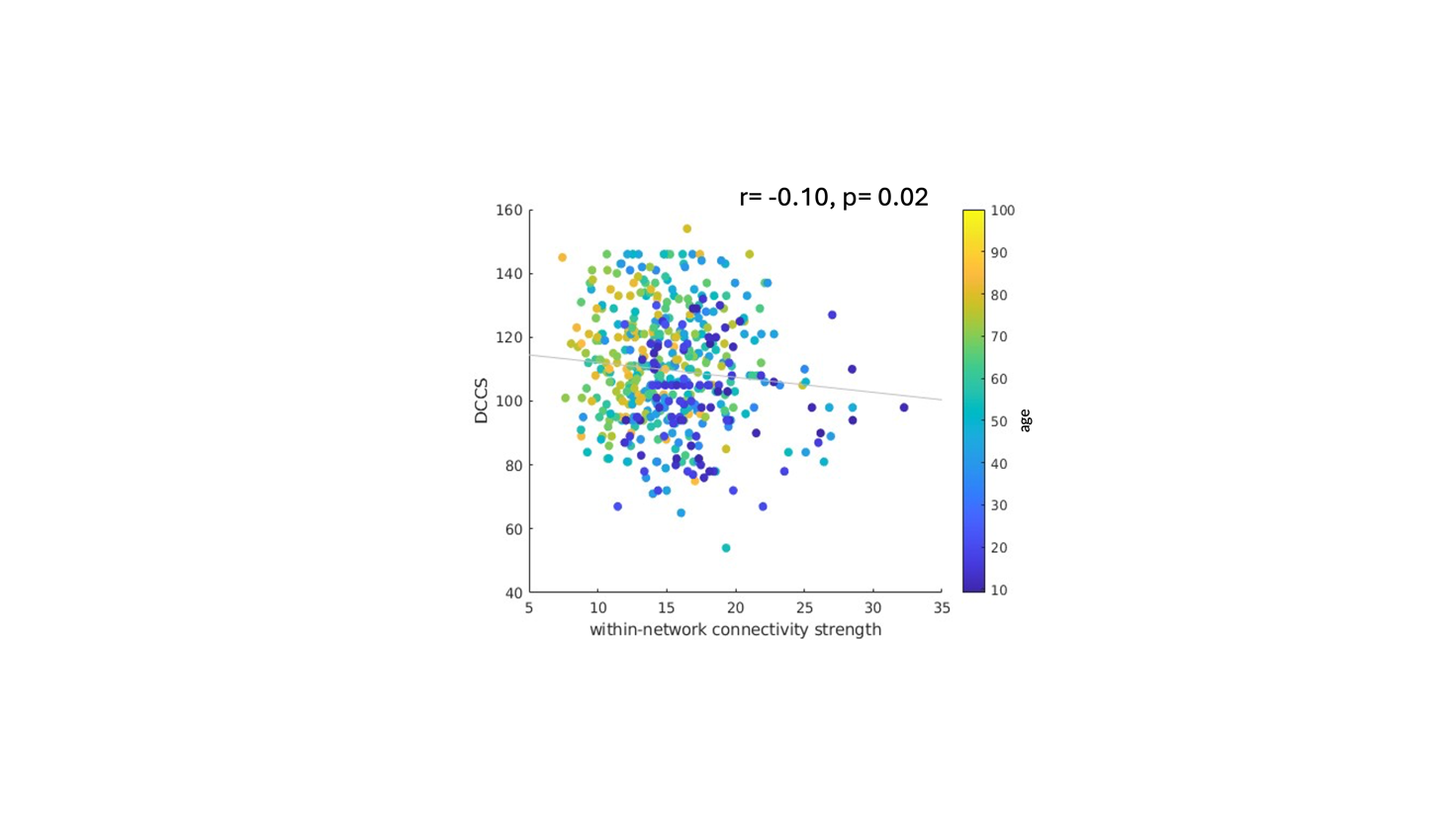


***Figure S5*.** **Within-network connectivity strength and EF performance.** A significant linear negative association was observed between within-network strength of the DMN and DCCS performance (r = -0.10, p = 0.02).
